# Thermally Actuated Soft Robotics

**DOI:** 10.1002/adma.202504683

**Published:** 2025-07-25

**Authors:** Shuang Wu, Seol‐Yee (Jennifer) Lee, Yong Zhu

**Affiliations:** ^1^ Department of Mechanical and Aerospace Engineering North Carolina State University Raleigh NC 27695 USA

**Keywords:** bioinspiration, soft actuators, soft robots, thermal actuation

## Abstract

Soft robots with exceptional adaptability and versatility have opened new possibilities for applications in complex and dynamic environments. Thermal actuation has emerged as a promising method among various actuation strategieis, offering distinct advantages such as programmability, light weight, low actuation voltage, and untethered operation. This review provides a comprehensive overview of soft thermal actuators, focusing on their heating mechanisms, material innovations, structural designs, and emerging applications. Heat generation mechanisms including Joule heating, electromagnetic induction, and electromagnetic radiation and heat transfer mechanisms such as fluid convection are discussed. Advances in materials are grouped into two areas: heating materials, primarily based on nanomaterials, and thermally responsive materials including hydrogels, liquid crystal elastomers, and shape‐memory polymers. Structural designs, such as extension, bending, twisting, and 3D deformable configurations, are explored for enabling complex and precise movements. Applications of soft thermal actuators span environmental exploration, gripping and manipulation, biomedical devices for rehabilitation and surgery, and interactive systems for virtual/augmented reality and therapy. The review concludes with an outlook on challenges and future directions, emphasizing the need for further improvement in speed, energy efficiency, and intelligent soft robotic systems. By bridging fundamental principles with cutting‐edge applications, this review aims to inspire further advancements in the field of thermally actuated soft robotics.

## Graphical abstract

This review focuses on the recent development of thermally actuated soft robotics, highlighting the four major heating mechanisms, as well as structural designs, thermal management, material innovations, and emerging applications. A summary and outlook section present the current challenges of thermally actuated soft robots and the future directions to address these challenges.

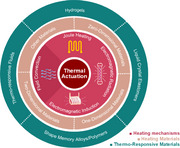

## 1 Introduction

In the realm of robotics, the quest for more versatile and adaptable mechanisms has led researchers to explore innovative avenues. Among these, soft robotics stands out as a promising field, offering advantages like better biocompatibility, delicate manipulation, environmental adaptability, and multi‐degree of freedom.^[^ ^1^, ^2^, ^3^, ^4^, ^5^, ^6^ ^]^ Soft robots have found wide applications in a variety of fields, including biomedical engineering, surgical assistance, active prosthetics, camouflage, and human‐machine interfaces.^[^ ^7^, ^8^, ^9^, ^10^, ^11^, ^12^ ^]^ To achieve versatile and adaptable actuation, researchers have explored different actuation methods for soft robots using a variety of stimuli, including pneumatic/hydraulic pressure,^[^ ^1^, ^13^, ^14^, ^15^, ^16^ ^]^ thermal,^[^ ^17^, ^18^, ^19^, ^20^ ^]^ electric field,^[^ ^21^, ^22^, ^23^, ^24^ ^]^ magnetic field,^[^ ^25^, ^26^, ^27^, ^28^ ^]^ and chemical potentials.^[^ ^29^, ^30^, ^31^ ^]^ Each actuation type offers distinct advantages and challenges, making them suitable for specific applications and environments. Among the various actuation methods explored in soft robotics, thermal actuation has garnered significant attention recently due to its unique combination of advantages, including structural simplicity, lightweight design, programmable thermal responsiveness, low actuation voltage requirements, absence of electrolytes or complex circuitry, and its strong potential for untethered operation in mobile or remote applications.^[^ ^32^, ^33^, ^34^, ^35^ ^]^

Pneumatic actuators typically offer fast and high‐force output but require precise sealing, bulky external compressors, and tubing. In comparison, thermal actuators are more easily miniaturized and integrated into compact systems. For example, a thermally actuated soft crawling robot can be fabricated with self‐weight below 1 g,^[^ ^36^, ^37^ ^]^ and less than 60 g with battery and control board included,^[^ ^38^ ^]^ which is challenging for pneumatic robot systems. Magnetic actuators can achieve remote and rapid actuation, but they generally rely on external magnetic fields and often require magnetic materials or coils, which add weight and complexity.^[^ ^39^ ^]^ Also, building a well‐programmed magnetic field with enough operation space has been challenging.^[^ ^40^ ^]^ In contrast, thermal actuators do not require external field‐generating equipment for control; instead, their actuation can be easily and locally managed in a programmable manner. The remote actuation dielectric elastomer actuators can deliver high‐speed, large‐strain actuation, but they typically require high driving voltages (400 to 9000 V)^[^ ^41^, ^42^ ^]^ and precise insulation to avoid dielectric breakdown, which limits their practical deployment.^[^ ^43^, ^44^ ^]^ In contrast, thermal actuators can be driven by low voltages (typically under 10 V),^[^ ^20^, ^34^ ^]^ allowing for safe and direct integration with flexible electronics. Chemically driven actuators, while capable of large deformations or bio‐inspired responses^[^ ^45^ ^]^ often suffer from slow actuation frequency (less than 0.01 Hz),^[^ ^46^ ^]^ poor repeatability, or challenges in avoiding environmental interference.^[^ ^47^ ^]^ While thermal actuation is sometimes limited by slower response and cooling times, ongoing advances in materials (e.g., built‐in cooling materials), thermal management strategies (e.g., embedded cooling, active convection), and structural designs (e.g., instability design) continue to address these limitations and improve the actuation frequency to close to 1 Hz.^[^ ^19^, ^48^ ^]^

Beyond the advantages outlined above, thermal soft robots hold strong potential for truly untethered operation. They can integrate their energy sources (e.g., batteries) and control components (such as printed circuit boards) directly with the actuation system. In contrast, other actuation methods—such as those relying on magnetic fields or electric fields—may appear untethered but in fact depend on complex, heavy, and bulky external equipment, which significantly restricts their range and functional space.^[^ ^42^, ^49^ ^]^

In general, two classes of heating mechanisms have been used for thermal actuation–heat generation within the functional materials, including Joule heating,^[^ ^17^, ^18^, ^20^, ^34^ ^]^ electromagnetic induction (EMI),^[^ ^50^, ^51^, ^52^ ^]^ and electromagnetic radiation (EMR) and heat transfer from the environment, such as fluid convection.^[^ ^53^, ^54^, ^55^ ^]^

The design of thermal actuators for soft robotics requires a delicate balance between material selection, structural design, and thermal control. Researchers have explored a myriad of materials with tunable thermal and mechanical properties, allowing for precise control over actuation behavior. Additionally, sophisticated structural designs and control systems have been developed to accomplish complex tasks and adapt to different environmental conditions.

The integration of thermal actuators into soft robotic systems has unlocked a variety of applications across various domains. In search and rescue, locomotion robots utilize thermal actuation to dynamically adapt their shape for movement across various terrains, offering enhanced mobility in uneven environments. In production lines, thermal soft grippers are adept at gripping delicate objects, making them suitable for applications in food handling, healthcare, and assembly tasks where precision and compliance are paramount. Inspired by nature, thermal actuation‐enabled biomimetic devices allow for innovative solutions in areas such as underwater exploration and environmental monitoring.

**Figure 1:**
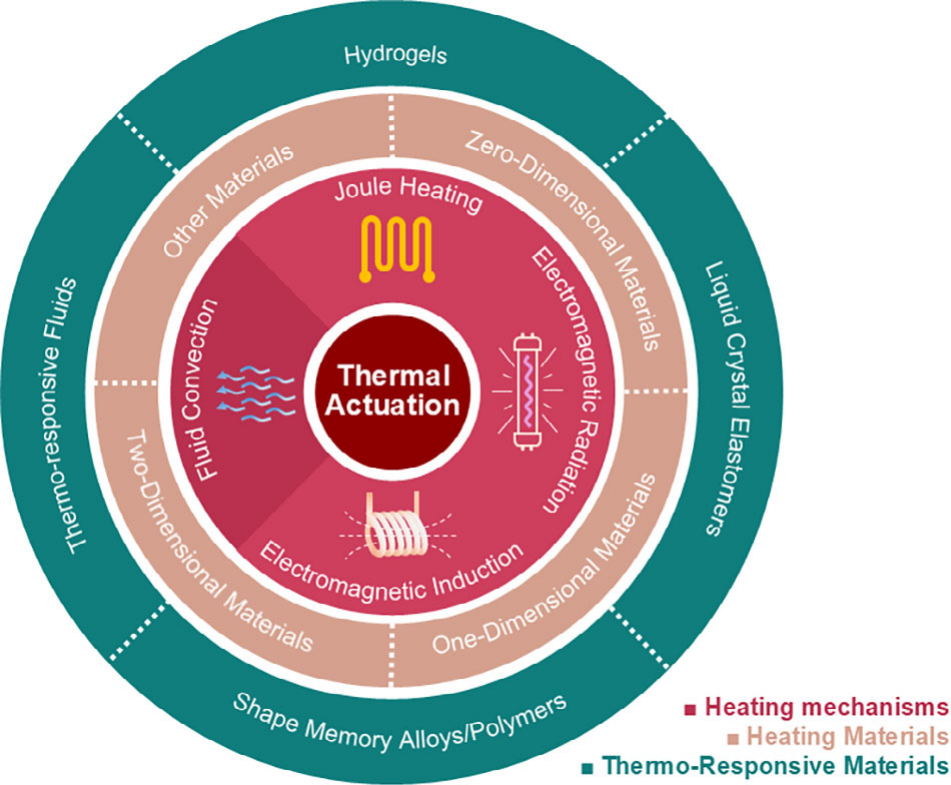
Overview of the thermal heating mechanisms and materials selection.

In this review, we aim to provide a summary of the latest developments in thermal actuators for soft robotics, highlighting the material selection, design considerations, applications, and future directions. As shown in **Figure** **1**, this review is organized as follows: In Section 2, we discuss the heating mechanisms. In Section 3, we summarize the heating materials, focusing on nanomaterials of different dimensions and thermally responsive materials. In Section 4, we highlight a variety of structural designs. In Section 5, we review the recent applications of thermally actuated soft robotics. By sharing insights from recent research endeavors, we hope to inspire further exploration and innovation in this exciting field, ultimately paving the way for the development of next‐generation soft robotic systems.

## 2 Heating Mechanisms

The prospects of soft robotics are closely tied to heating mechanisms and the development of smart materials and structural designs.^[^ ^43^, ^56^, ^57^, ^58^, ^59^, ^60^, ^61^, ^62^, ^63^, ^64^, ^65^, ^66^, ^67^, ^68^ ^]^ This section provides an overview and classification of different thermal actuation methods, highlights key research in each area, and outlines the relevant performance metrics.

### 2.1 Heating Mechanisms

Thermal actuation refers to the transduction process through which thermal energy is converted into mechanical energy. This conversion can happen in two ways: heat generation and heat transfer. Heat can be applied directly through thermal conduction to raise the temperature of the target materials. Alternatively, heat may be generated within the functional materials and subsequently transferred to adjacent components. In the context of soft robotics, heat transfer typically involves a fluidic medium that facilitates heat conduction; in our work, we represent this form of heat transfer through fluid convection. For heat generation, we categorize the mechanisms into three main types based on their energy input modes: Joule heating, EMI, and EMR.

Joule heating is typically realized in tethered systems, where the actuator is connected to an external power source. The two electromagnetic methods (EMI and EMR) are untethered, with some requiring external equipment such as an electromagnet or a light source. Similarly, fluid convection can mostly be considered untethered but requires an external heat source and thermal conductive media. Untethered robotic systems can be actuated via remote and contactless control, broadening their potential applications. For the electromagnetic heating mechanisms, the soft robot's operation space, position, and shape are highly dependent on the externally applied magnetic field and the actuator's relative position to the source. Joule heating can be untethered by the integration of computational units, power systems, and wireless modules, but the integration between the soft body and some rigid components remains challenging.^[^ ^63^, ^73^ ^]^

#### 2.1.1 Joule Heating

Joule heating is the most widely adopted thermal actuation method in soft robotics. It operates by converting electrical energy into heat as current passes through a resistive material, where the material's inherent electrical resistance generates thermal energy.

Joule heating allows for controlled and localized heating, which can be realized via patterning of the conductive layer. This method is useful to heat materials like shape‐memory polymers/alloys and hydrogels, which require careful temperature regulation to avoid degradation.^[^ ^58^, ^60^ ^]^ Joule heating allows for fine‐tuning of the temperature to stay within these critical thresholds, making it ideal for programming and controlling the actuation of soft robots and actuators. By adjusting the current through or the resistance of the conductive material, one can ensure that the material's temperature remains within the desired range, optimizing the performance of the actuator and preventing damage.

**Figure 2:**
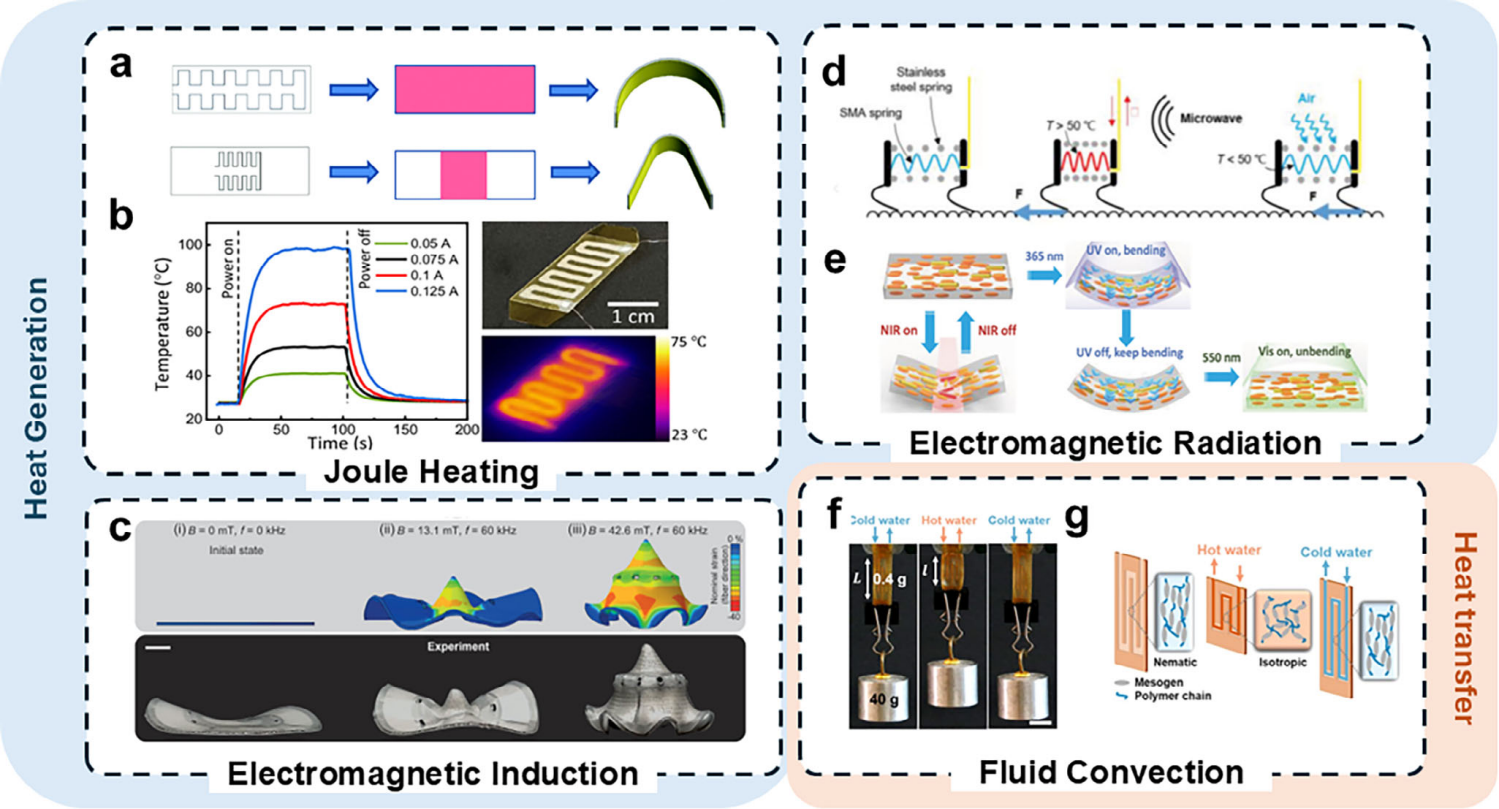
Four different heating mechanisms for thermal actuation. a) Schematic of conductive patterns, the approximate areas that will be heated upon actuation, and the resulting shape change of a bimorph bending actuator (left to right) (Reproduced with permission.^[^ ^69^ ^]^ Copyright 2021, Royal Society of Chemistry). b) A thermal bimorph actuator for a Kresling Origami unit: temperature as a function of time for different currents applied from 0.05 to 0.125 A, a photograph and an IR image of the patterned heater (Reproduced with permission.^[^ ^20^ ^]^ Copyright 2023, American Association for the Advancement of Science). c) Simulation and experimental images of LCE‐LM actuator with different EMI frequencies and magnitudes (Reproduced with permission.^[^ ^52^ ^]^ Copyright 2023, Wiley‐VCH). d) Schematic of a SMA‐based quadruped crawler actuated by microwave radiation (Reproduced with permission.^[^ ^70^ ^]^ Copyright 2022, Wiley‐VCH). e) Schematic of a photothermal LCE actuator with the actuation behavior dependent on the applied wavelengths. NIR causes reversible local deformation, UV creates permanent bending, and visible light reverses the bending induced by the UV light (Reproduced with permission.^[^ ^71^ ^]^ Copyright 2018, Wiley‐VCH). f) LCE‐based actuator with an applied heating and cooling cycle lifting and releasing a 40 g weight, respectively. g) Schematic of an LCE‐based actuator with fluid convection‐based heating and cooling cycles (f and g: Reproduced with permission.^[^ ^72^ ^]^ Copyright 2020, American Chemical Society).

Exploiting this precision has allowed for programmable deformation of bimorph structures: uniform bending, customized bending, folding, and twisting (**Figure** **2**a).^[^ ^20^, ^34^, ^69^ ^]^ Building upon this, Wu et al. have developed caterpillar‐inspired soft crawling robots, where specific sections of the crawler are heated for localized actuation and controlled bending curvature for forward and backward locomotion.^[^ ^20^ ^]^ To achieve a more challenging steering motion for thermally actuated soft robots, Wu et al. demonstrated electrothermal actuators with more complex robot designs. A modular Kresling Origami robot was developed to crawl forward, backward, and steer with programmed trajectories. Figure 2b demonstrates the electrothermal actuation of a folding actuator for a single Kresling unit.^[^ ^34^ ^]^ The programmability of Joule heating facilitates sequential and cyclic actuation of soft robots.

While Joule heating offers precise and localized temperature control, it comes with certain drawbacks. Notably, Joule heating has relatively low actuation efficiency and low frequency.^[^ ^4^, ^63^, ^65^ ^]^ A significant portion of the energy is wasted due to the energy being dissipated and wasted during the cooling process.^[^ ^65^ ^]^ The low frequency is due to relatively slow heating and cooling cycles in the soft materials. Electrothermal actuation requires a conductive layer, which affects the overall functional strain range of the soft actuator and reduces the recyclability of the actuator. The coupling of materials between the heating element and the functional material increases the total mass of the actuator, compromising efficiency and power‐to‐mass output.^[^ ^73^ ^]^ Another challenge of electrothermal actuation is the untethered operation. Electric wiring is generally required to provide the power, except for portable power sources such as batteries.

#### 2.1.2 Electromagnetic Induction

EMI is the process by which a changing magnetic field induces an electric current in a conductive material. When the conductive materials, such as metals or conductive polymers, are exposed to an alternating magnetic field, eddy currents are generated that produce resistive heating and raise the temperature of the materials. In soft robotics, this heat can be harnessed to actuate thermally responsive materials, causing them to deform in a controlled manner. Researchers have developed EMI‐based soft robots that crawl like a larva,^[^ ^36^ ^]^ contract like muscles,^[^ ^74^ ^]^ and walk like turtle fins.^[^ ^52^ ^]^

A common way of using EMI for soft robotics is through wireless power transmission. A wireless power transmission system is composed of a pair of charging and receiving coils. The charging coil generates varying electromagnetic fields and induces an eddy current in the receiving coil. Instead of causing the receiving coil itself to heat up, the circuit with the eddy current is connected to external devices. For example, Zhang et al. developed a robot with EMI‐induced power transfer in implantable systems, particularly in enclosed environments such as the human body.^[^ ^75^ ^]^ The device incorporated a tri‐layered structure – a thermally responsive poly(N‐isopropylacrylamide) (PNIPAAm) hydrogel layer, a layer with silver nanowire (AgNW) heater and a copper coil, and a polyimide (PI) layer. The copper coil was for wireless power transmission, which supplies current to the AgNW heater. The PNIPAAm hydrogel and the PI layer formed the thermal bimorph. The system allowed for a stable power supply of ≈1.05 W at 15 MHz with an efficiency of 80%, generating sufficient power to trigger the actuation of the soft robot.

EMI‐based actuation has gained attention for its true untethered actuation. Maurin et al. developed a liquid crystal elastomer (LCE)‐liquid metal (LM) composite thermal actuator for untethered underwater actuation.^[^ ^52^ ^]^ Figure 2c shows the sequential actuation (thermo‐structural finite element analysis simulations and experiments) under the three different magnetic field amplitudes. In addition to untethered actuation, this design also took advantage of the room‐temperature water to facilitate rapid cooling to enhance the response speed.

There are two primary ways for applying an alternating magnetic field–one way involves using an electromagnet, which requires a very limited working space in order to generate strong and uniform magnetic fields, while the other uses a portable charging coil that generates a middle‐concentrated but gradually diverging magnetic field. For the portable coil, controlling the intensity and direction of the generated magnetic field can be difficult, requiring precise movement of the coil, e.g., using robotic arms.^[^ ^76^ ^]^ EMI can achieve distributed heating. Conductive materials can be patterned on a substrate, so the eddy current can be confined in the patterned materials, heating up the pattern and the neighboring area. However, within the conductive pattern, it is difficult to achieve programmable heating (e.g., heating selected parts of the pattern), as the magnetic fields tend to take affect the entire area.^[^ ^51^ ^]^

#### 2.1.3 Electromagnetic Radiation

EMR encompasses a broad spectrum of wavelengths, including radio waves, microwaves, infrared (IR), visible light, ultraviolet (UV), X‐rays, and gamma rays (in the order of decreasing wavelength). The choice of wavelength for heating soft robots depends on the specific application, material properties, and desired actuation performances. IR, near‐infrared (NIR), and visible lights are widely adopted for soft robotic actuation due to their balance of efficiency, precision, and safety. Radio waves and microwaves are suitable for deeper penetration, yet with lower energy. UV actuation is ideal for high‐precision tasks but is limited by shallow penetration. X‐rays and gamma rays, while powerful, are generally impractical due to safety and complexity concerns.

In the context of thermal actuation, EMR heats materials through photon‐driven energy transfer. Because photons carry energy, momentum, and angular momentum, when these photons interact with the target material, they excite the material's molecular structure, causing it to vibrate. The resulting vibrational motion leads to increased kinetic energy, manifesting as heat. All EMR heating mechanisms can be described as thermal excitation, with energy transfer occurring via molecular vibrations or dipolar reorientation, depending on wavelength and material properties. These interactions are governed by quantum transitions in molecular energy levels, which, when followed by non‐radiative relaxation, lead to localized heating.

Particularly, dielectric heating, relevant at microwave and radio frequencies, relies on the rapid rotation of polar molecules with an electric dipole moment in response to the oscillating electric field. These molecules rotate back and forth rapidly, encountering resistance from surrounding molecules and dissipating energy in the form of heat. This process includes Brownian heating, where physical rotation or oscillation of nanoparticles within a surrounding fluid creates forces that convert electromagnetic energy into thermal energy. Unlike photothermal heating, which depends on photon absorption by chromophores, dielectric heating results from the bulk polarization of the material and the mechanical friction induced by particle rotation.

Several innovative soft robotic devices have been developed using EMR‐based thermal actuation. For instance, researchers have created light‐driven artificial muscles using LCEs, where localized NIR irradiation induces shrinkage in LCE fibers.^[^ ^77^ ^]^ Similarly, photothermal‐responsive hydrogels have been used to fabricate soft walkers that move in response to patterned light exposure.^[^ ^78^ ^]^

EMR‐based thermal actuation offers several advantages over other mechanisms. Compared to electrothermal actuation, EMR enables untethered actuation, enhancing the adaptability of soft robots. Also, EMR can fine‐tune the spatial distribution of thermal input without the need for embedding patterned conductive fillers. Figure 2d shows an untethered quadruped crawling robot composed of a shape memory alloy (SMA) spring.^[^ ^70^ ^]^ Figure 2e shows a reprogrammable LCE‐based soft actuator that is designed to be responsive to both UV and visible light.^[^ ^71^ ^]^ Compared to EMI, EMR required much simpler equipment for operation. Compared to fluid convection, EMR shows much better control, both spatial and temporal. A light‐driven, carbon‐based composite robot showed several locomotion modes, including forward, rolling, and steering motion by controlling the distribution of light.^[^ ^79^ ^]^

However, EMR‐enabled actuation also has its limitations. The penetration depth of radiation can be restricted to certain materials, and the efficiency of energy conversion depends on the absorption properties of the thermally responsive material. Additionally, precise control of radiation intensity and distribution is necessary to avoid overheating or uneven heating. Another limitation of EMR actuation is that EMR typically propagates along a straight line, which can cause the deformed actuator to obstruct the EMR light source. This feature of self‐obstruction in EMR heating is discussed in the work of Zhao et al.,^[^ ^80^ ^]^ where they developed photothermal LCE/elastomer bilayer oscillators. In comparison to Joule heating or EMI actuation, EMR actuation is less suitable for structures that involve folding, rolling, or overlapping, such as Origami‐based structures.

#### 2.1.4 Heat Transfer

Convection or convective heat transfer is the process of heat transfer through the movement of fluids (liquids or gases) caused by differences in temperature and density within the fluid. Convective heat transfer provides a unique advantage in fast cooling of the thermal actuators, which is often challenging for other thermal actuation methods. The use of heating and cooling cycles improves the speed of thermal actuators. Convective heat transfer also simplifies the structural design, contrary to methods such as Joule heating that require the integration of conductive materials. An example of this mechanism is a fluid‐driven LCE actuator that generated a large reversible strain, 15%, at 0.17 Hz (Figure 2f).^[^ ^72^ ^]^ The schematic of the internal channels can be seen in Figure 2g. Similarly, a work on artificial muscle employed fluid convective as a proof‐of‐concept for implementing convective cooling, effectively reducing cooling times without the addition of extra materials to the device.^[^ ^81^ ^]^ As a result, high actuation frequencies (1 Hz) at 12% reversible actuation with 8.4 MPa load were demonstrated.

Another benefit of convective heat transfer is its ability to effectively regulate the hydration levels of hydrogels during actuation cycles and maintain more uniform temperature distribution throughout the hydrogel. Bulk hydrogel often faces challenges with uniform heating when using traditional conductive heating, as the surfaces of the hydrogel in contact with the heating method deform faster than the inner bulk. The uneven deformation creates mechanical constraints that limit the deswelling rate. In contrast, immersing the hydrogel in a bulk fluid enables uniform heating and cooling as it allows the hydrogel to absorb and release fluids at a more consistent deswelling/swelling rate.

While certain thermal actuators operate more efficiently with convective heat transfer, convective heat transfer presents a challenge for underwater or in vivo thermal actuation, as surrounding fluid temperatures can complicate temperature control and actuation efficiency. However, this challenge can be leveraged as an advantage. Ongaro et al. utilized body temperature by programming the grasping motion of a micro‐robot to activate when the water bath reached 37 °C.^[^ ^82^ ^]^ Another work leveraged in vivo body temperature by engineering cardiovascular stents that can be compressed to a reduced size for delivery at room temperature and then expanded to a pre‐programmed size upon reaching internal body temperature.^[^ ^83^ ^]^

### 2.2 Thermal Management

**Figure 3:**
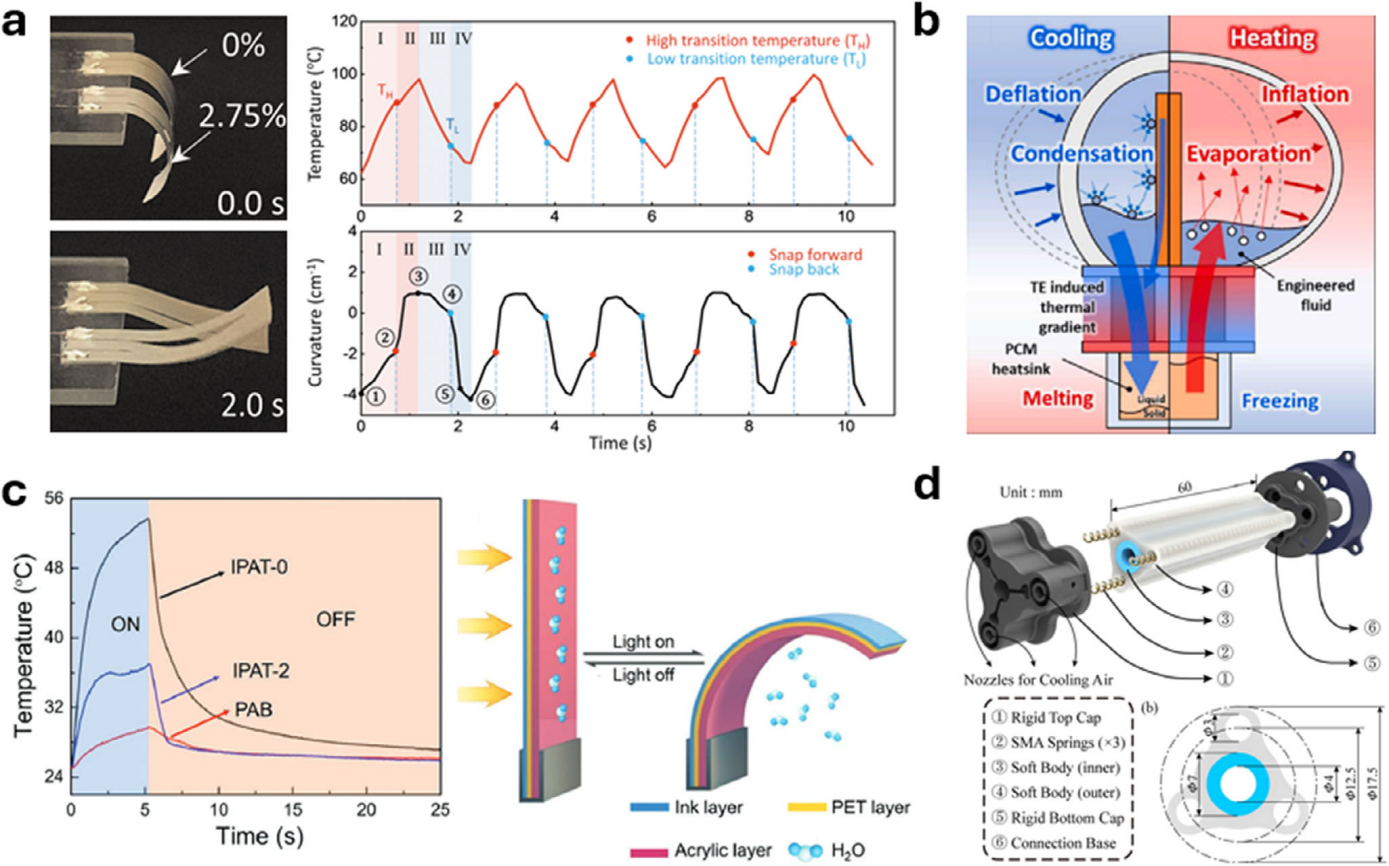
Thermal management for soft thermal actuators. a) A soft bimorph actuator with self‐sensing function. The real time temperature feedback facilitates the high frequency snap‐through and snap‐back (Reproduced with permission.^[^ ^19^ ^]^ Copyright 2022, Mary Ann Liebert Inc). b) A bioinspired untethered soft robot employing pumpless phase change soft actuators powered by two‐way thermoelectrics (Reproduced with permission.^[^ ^84^ ^]^ Copyright 2023, Elsevier). c) A photothermal bimorph actuator equipped with an in‐built cooling layer (Reproduced with permission.^[^ ^48^ ^]^ Copyright 2019, Wiley‐VCH). d) Active‐cooling‐in‐the‐loop controller for an SMA‐driven soft robotic tentacle (Reproduced with permission.^[^ ^85^ ^]^ Copyright 2023, IEEE).

Thermal management of thermally actuated soft robots is an essential aspect that influences their controllability and efficiency. One prevalent approach to enhancing thermal management of soft robots is concurrent temperature sensing, which facilitates not only quick response time but also optimal performance. The integration of temperature sensors with thermal actuators allows for real‐time monitoring of temperature distribution, enabling soft robots to adapt their performance according to varying environmental temperatures and maintain desired operating conditions. **Figure** **3**a shows a soft bimorph actuator with self‐sensing function enabled by the use of the AgNW heater,^[^ ^19^ ^]^ which can monitor temperature based on its resistance change. T_H_ and T_L_ represent the critical temperatures for snap‐through (snap‐forward) and snap‐back, respectively. The key is to turn off the heater shortly after the temperature goes above T_H_ (i.e., snap‐through has occurred) and similarly turn on the heater shortly after the temperature goes below T_L_ (i.e., snap‐back has occurred). So, the actuator is operated essentially between the snap‐through and snap‐back, increasing the overall speed of the actuator. Temperature sensing is critical to provide real‐time feedback here. In addition to increasing the speed, such feedback based on the temperature sensing can prevent overheating, which can lead to material degradation and functional failure in soft robots.

Active cooling also plays an important role in the thermal management of soft robots, improving their actuation frequency. One innovative example is a soft thermo‐pneumatic actuating module employing the thermoelectric effect and liquid‐gas phase transition to generate pneumatic pressure^[^ ^84^ ^]^ (Figure 3b). Of note is the thermoelectric cooling effect exhibited a much faster deflation rate compared to natural convective cooling, therefore shortening each actuating cycle and substantially improving the actuation frequency. This work demonstrated a soft gripper and an autonomous untethered bio‐inspired crawling robot with significant improvement in speed compared to other phase change material‐based robots.

Another noteworthy approach is the development of photothermal bimorph actuators that come equipped with built‐in cooling solutions for applications such as light mills, frequency switches, and soft robots^[^ ^48^ ^]^ (Figure 3c). This system utilized photothermal effects for thermal actuation while simultaneously employing an additional acrylic layer to cool down the actuator. After a certain time of heating, the water within the acrylic layer starts to evaporate and absorb heat to cool down the actuator. This process greatly reduces the recovery time of the actuator.

Finally, a more universal approach is to use external sensors and printed circuit boards to provide feedback control and actively cool down the soft actuators. Researchers have reported a soft tentacle‐like robotic arm actuated by SMA actuators.^[^ ^85^ ^]^ The actuators are integrated with an air blowing cooling system, which responds to computer vision sensing results (Figure 3d). This way of thermal management can be applied to a variety of different heating mechanisms and sensing mechanisms. The major limitations of this approach are the requirement of external sensing devices and a relatively complicated control system.

## 3 Materials for Soft Thermal Actuation

### 3.1 Heating Materials

Soft thermal actuators and thermally actuated soft robots harness the power of heat to induce deformation and motion in soft materials. The choice of materials plays a critical role in determining performance, responsiveness, and efficiency. In this section, we present a diverse array of soft heating materials that can generate heat under various heating mechanisms as delineated in Section 2.1, from nanomaterials to macro scaled materials, and discuss their unique properties, advantages, and limitations.

Among all the heating materials, a major class is nanomaterials that are usually embedded in soft matrices to form soft heaters. Nanomaterials, including 0D, 1D, 2D nanomaterials, are widely used for Joule heating and EMR. Unlike conventional metal wires/sheets,^[^ ^18^, ^86^ ^]^ nanomaterials, typically in the form of a percolation network, show a great potential for providing uniform, programmable, and biocompatible thermal actuation when integrated with soft matrix materials. For EMI, both nanomaterials and bulk conductive materials have been used, while fluid convection is mainly enabled by bulk media like water and air. When selecting heating materials for the different heating mechanisms, several key considerations include mechanical flexibility and stretchability, electrical conductivity, thermal conductivity, thermal stability, and photothermal conversion efficiency.

#### 3.1.1 0D Nanomaterials

**Figure 4:**
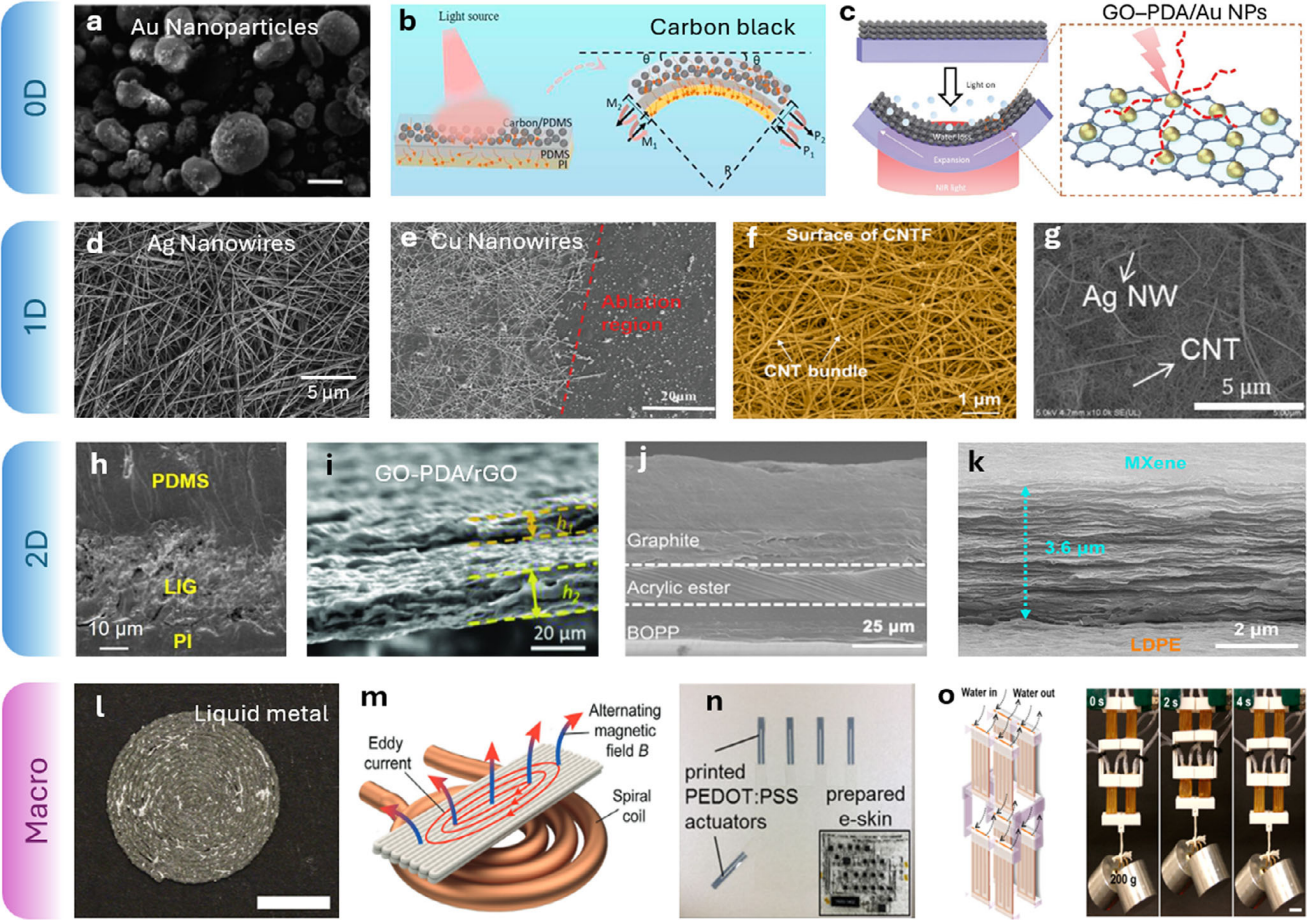
Heating materials for thermally actuated soft robots. a) Scanning electron microscope (SEM) image of AuNPs coated with shells (Reproduced with permission.^[^ ^87^ ^]^ Copyright 2021, Wiley‐VCH). The scale bar is 20 microns. b) Schematic representation of CB‐based actuators with a sandwich structure under UV light (Reproduced with permission.^[^ ^88^ ^]^ Copyright 2022, Elsevier). c) Actuation mechanism of a GO‐polydopamine/AuNPs bilayer structure, which integrates thermal expansion and desorption during light exposure (Reproduced with permission.^[^ ^79^ ^]^ Copyright 2020, Wiley‐VCH). d) SEM image of AgNW network (Reproduced with permission.^[^ ^89^ ^]^ Copyright 2024, Royal Society of Chemistry). e) CuNWs patterned by UV laser ablation method on top of polyethylene terephthalate (Reproduced with permission.^[^ ^90^ ^]^ Copyright 2022, Wiley‐VCH). f) SEM image of the CNT film (Reproduced with permission.^[^ ^91^ ^]^ Copyright 2023, American Chemical Society). g) SEM images of the AgNW/CNT‐coated conductive fabric (Reproduced with permission.^[^ ^92^ ^]^ Copyright 2020, Wiley‐VCH). h) The cross‐section SEM image of LIG based actuator (Reproduced with permission.^[^ ^93^ ^]^ Copyright 2020, Wiley‐VCH). i) Composite film was fabricated with GO‐polydopamine /reduced graphene oxide layer (Reproduced with permission.^[^ ^94^ ^]^ Copyright 2019, Wiley‐VCH). j) SEM image showing the cross‐sectional view of the graphite enabled actuator (Reproduced with permission.^[^ ^95^ ^]^ Copyright 2024, Wiley‐VCH). k) Cross‐sectional SEM images of MXene/LDPE bilayer film (Reproduced with permission.^[^ ^96^ ^]^ Copyright 2021, American Chemical Society). l) 4D printed LM/LCE in a spiral pattern (Reproduced with permission.^[^ ^97^ ^]^ Copyright 2020, American Chemical Society). Scale bar is 5 mm. (m) Schematic of printed LM film under EMI heating (Reproduced with permission.^[^ ^52^ ^]^ Copyright 2023, Wiley‐VCH). n) A robotic hand with five fingers made of PEDOT: PSS/PDMS actuators (Reproduced with permission.^[^ ^98^ ^]^ Copyright 2018, Mary Ann Liebert Inc). o) A group of LCE actuators with running hot/cold water in the built‐in micro channels (Reproduced with permission.^[^ ^72^ ^]^ Copyright 2020, American Chemical Society).

0D nanomaterials, including metal nanoparticles (NPs),^[^ ^79^, ^87^, ^99^ ^]^ carbon‐based NPs,^[^ ^88^ ^]^ and other conductive NPs,^[^ ^100^ ^]^ offer unique opportunities for thermal actuation (**Figure** **4**a–c). By dispersing the NPs within soft matrices, researchers can fabricate thermally responsive composites capable of generating localized heating and inducing controlled deformation.^[^ ^101^ ^]^ Metal NPs, such as silver NPs (AgNPs) and gold NPs (AuNPs), exhibit high thermal conductivity, enabling efficient heat transfer and hence rapid actuation in soft thermal actuators.^[^ ^102^, ^103^ ^]^ According to the percolation theory,^[^ ^104^, ^105^ ^]^ for a given network density, the electrical conductivity increases with the length of the conductor. Thus, compared to 1D and 2D nanomaterials, the percolation network of 0D nanomaterials typically shows lower electrical conductivity. However, NPs have a higher surface area relative to their volume, which allows them to absorb light more efficiently compared to 1D and 2D materials. Metal NPs, like AuNPs and AgNPs, can exhibit a phenomenon known as localized surface plasmon resonance, where conduction electrons oscillate in resonance with incident light.^[^ ^106^ ^]^ This effect further enhances photothermal conversion, making it more suitable for EMR. Figure 4c shows a bilayer composite film made of GO‐DA‐AuNPs/PDMS. The plasmonic effect of AuNPs greatly facilitated the EMR and photothermal‐induced bending of the composite film.^[^ ^79^ ^]^

Other than metal NPs, Carbon‐based NPs, such as carbon black (CB),^[^ ^88^ ^]^ also show good thermal conductivity and photothermal conversion efficiency. CB composites show the highest photothermal conversion efficiency in the IR region of the electromagnetic spectrum. By mixing CB with PDMS resin and then laminating with PI film, researchers fabricated a mechanical gripper and a bionic flower‐like robot that operated using photothermal actuation.^[^ ^88^ ^]^ Moreover, the lightweight and scalable nature of carbon‐based NPs facilitates the fabrication of soft thermal actuators.

#### 3.1.2 1D Nanomaterials

1D nanomaterials, such as metal nanowires (NWs)^[^ ^17^, ^20^, ^34^, ^90^ ^]^ and carbon nanotubes (CNTs),^[^ ^91^, ^107^ ^]^ show great potential for thermal actuators and soft robots (Figure 4d–g). Metal NWs, such as AgNWs^[^ ^17^, ^108^ ^]^ and copper NWs (CuNWs),^[^ ^90^ ^]^ exhibit high aspect ratios and electrical conductivity, enabling efficient Joule heating. Compared with 0D nanomaterials, the percolation network formed by 1D nanomaterials in a polymer matrix offers a higher level of intrinsic stretchability, which is of great importance for soft robotic applications. By embedding metal NWs into elastomeric matrices, researchers can fabricate flexible and stretchable heaters capable of generating uniform and fast heating. With printing or other patterning techniques, patterned metal NWs can precisely define the heating areas and achieve controlled localized deformation.^[^ ^20^ ^]^

CNTs are composed of cylindrical structures of carbon atoms, exhibiting exceptional thermal conductivity (6600 W m^−1^ K^−1^), electrical conductivity (10^6^ S m^−1^), and mechanical strength (1.8 TPa), making them excellent candidates for thermal actuators and soft robots.^[^ ^109^ ^]^ More importantly, CNTs exhibit not only good electrical conductivity but also photothermal properties because of their unique ability to absorb light, particularly in the NIR region. By mixing CNTs within polymer matrices, researchers have fabricated thermally responsive composites capable of not only efficient Joule heating but also EMR heating.^[^ ^91^, ^107^, ^110^, ^111^ ^]^ Figure 4f shows a layered CNT/PDMS composite film with low density and a large number of pores, whose interior is full of air. Through Joule heating‐triggered air expansion, the layered CNT composite film showed fast response and large output force (actuator expanded in 2.9 s lifting up to ≈50 times its weight).^[^ ^91^ ^]^ For photothermal actuation, an LCE‐CNT composite‐based soft robot was developed with multi‐modal inchworm‐like locomotion modes (crawling, squeezing, and jumping) by tailoring the light scanning patterns.^[^ ^112^ ^]^

#### 3.1.3 2D Nanomaterials

2D nanomaterials, such as graphene,^[^ ^94^, ^113^ ^]^ graphene oxide (GO),^[^ ^79^, ^114^ ^]^ MXene,^[^ ^96^ ^]^ and transition metal dichalcogenides,^[^ ^115^ ^]^ also exhibit great potential for thermal actuators and soft robots (Figure 4h–k). Graphene, a single layer of carbon atoms arranged in a hexagonal lattice, exhibits exceptional thermal conductivity, mechanical strength, and electrical conductivity, making it an outstanding candidate for electrothermal actuators.^[^ ^116^ ^]^ By integrating graphene‐based heating elements into soft actuators, researchers can achieve rapid and efficient joule heating. A laser‐induced graphene (LIG) based thermal actuator has been used to demonstrate an artificial muscle that can lift a mass ≈110 times their weight in 3 s.^[^ ^93^ ^]^

MXene is a family of 2D inorganic compounds obtained by selectively etching specific atomic layers from multiple‐layered carbon (nitrogen) compounds. MXene is intrinsically hydrophilic, and yet, it has shown higher electrical conductivity than solution‐processed graphene.^[^ ^117^ ^]^ Researchers have fabricated a Ti_3_C_2_T_x_ MXene/low‐density polyethylene (LDPE) bilayer actuator by spraying an aqueous dispersion of MXenes onto a plasma‐activated LDPE film.^[^ ^96^ ^]^ The actuator can be programmed for biomimetic applications due to the eminent light absorption and photothermal/electrothermal responsiveness of MXenes.

2D nanomaterials, strictly speaking, are those with a single atomic layer. There are other nanomaterials with a thickness on the order of nanometers but much larger in‐plane dimensions, which can be called 2D‐like nanomaterials, such as nanoflakes, nanomembranes, or nanofilms. Nanoflakes, including metal nanoflakes^[^ ^118^ ^]^ and molybdenum disulfide (MoS_2_) flakes,^[^ ^119^ ^]^ show high aspect ratios and tunable thermal conductivity. By dispersing MoS_2_ nanoflakes within polymer matrices, researchers have fabricated a thermally responsive actuator. The fabricated actuator exhibited fast thermomechanical response, high reversibility, and long cycle life.^[^ ^119^ ^]^

#### 3.1.4 Other Materials

In addition to 0D, 1D, and 2D nanomaterials, other materials like metal wires,^[^ ^18^, ^86^ ^]^ LMs,^[^ ^52^, ^97^ ^]^ conductive polymers,^[^ ^98^ ^]^ organic dyes^[^ ^120^ ^]^ and pigments^[^ ^121^ ^]^ also offer unique functionalities for thermal actuators and soft robots.

LMs have been used for Joule heating, EMI, and EMR heating. Ambulo et al. introduced a printed composite ink composed of an LCE matrix with dispersed droplets of EGaIn, a commonly used type of LMs (Figure 4l). At low LM concentrations, the composite actuator exhibited a photothermal response upon near‐IR light. At higher LM concentrations, the embedded LM droplets can form percolating networks for the Joule heating of the LCE.^[^ ^97^ ^]^ Another work developed an LCE/LM composite (Figure 4m) leveraging the eddy current induction heating, which enabled ultrafast and programmable actuation.^[^ ^52^ ^]^

Conductive polymers, such as polyaniline and poly(3,4‐ethylenedioxythiophene) (PEDOT), exhibit tunable mechanical properties, mixed ionic and electronic conductivity. Combining PEDOT and PSS (polystyrene sulfonate) with advanced 3D printing has shown unprecedented opportunities in soft electronics and soft robotics.^[^ ^98^ ^]^ By doping conductive polymers with dopants such as acids or ions, researchers can tune their electrical conductivity and thermal stability, facilitating the fabrication of thermally responsive actuators for soft robotics.^[^ ^122^ ^]^ Figure 4n shows a wirelessly actuated robotic hand enabled by poly(3,4‐ethylenedioxythiophene) polystyrene sulfonate (PEDOT:PSS)/PDMS bilayer electrothermal actuators.^[^ ^98^ ^]^

Researchers developed NIR‐responsive vitrimers by incorporating a carboxyl‐bearing croconaine dye (CR‐800) as a photothermal agent. These CR‐vitrimers exhibited dynamic covalent bonding properties, allowing for the fabrication of hand‐shaped actuators with “fingers” that had different NIR‐response threshold values.^[^ ^120^ ^]^ Under NIR laser irradiation (λ = 808 nm), the actuators demonstrated selective and programmable movements, showcasing the potential of croconaine dyes in photothermal soft robotics applications. This example highlights how specific dyes can be utilized to achieve controlled and responsive actuation in soft robotic systems through photothermal effects. Another study presents photothermal soft actuators inspired by cuttlefish, where embedded NIR‐absorbing dyes enable light‐driven in‐plane bending and 3D shape morphing in cholesteric liquid crystal elastomer films.^[^ ^121^ ^]^ These actuators also feature temperature‐responsive dye aggregation, allowing synchronized changes in both shape and color, demonstrating potential for adaptive camouflage and signaling in soft robotics.

For fluid convention, a large variety of bulk materials, such as water and air, have been adopted as heat sources/media to transfer thermal energy. Figure 4o shows an example of a fluid channel‐enabled thermal actuator. The actuation and relaxation are enabled by running hot/cold water through the embedded channels inside LCE.^[^ ^72^ ^]^

### 3.2 Thermally Responsive Materials

Soft robots are typically made from materials with a wide range of Young's moduli (10^2^–10⁹ Pa), encompassing extremely soft hydrogels and silicone elastomers as well as stiffer materials such as nylon or polylactic acid, depending on the application.^[^ ^3^ ^]^ The low modulus of these materials leads to high compliance, allowing soft robots to conform to the surfaces of objects or obstacles. This property is crucial for applications requiring adaptable and gentle interaction with irregular or delicate surfaces, such as grasping fragile objects or navigating through confined spaces. Additionally, these materials exhibit high impact absorption due to their ability to deform and store energy during collisions, while their low contact pressure reduces the risk of damage to both the robot and its environment.^[^ ^3^, ^130^, ^131^ ^]^

Soft thermal actuators and robots must precisely control both movement and force to adapt to tasks across scales from microscale to macroscale. To achieve optimal control, researchers are exploring smart thermally responsive materials under various environmental conditions and temperature ranges. This section focuses on the characteristics and applications of several important classes of functional materials, including hydrogels, LCEs, SMAs/SMPs, and thermally responsive fluids. These materials are selected based on their thermal properties, tunability, and reversibility. For example, thermally responsive hydrogels exhibit intelligent responses such as volumetric changes, self‐healing, or controlled permeability. LCEs have demonstrated rapid actuation speeds and tunable anisotropic mechanical properties. SMPs exhibit programmable shape switching, enabling them to transition from one or more temporary shapes back to their original form when actuated. However, challenges such as managing hysteresis, increasing actuation speed, and understanding the balance between intrinsic properties and structural designs highlight the need for further research and development.

#### 3.2.1 Hydrogels

Hydrogel is a 3D hydrophilic polymer network that can retain a large amount of water or aqueous fluids within its structure (often over 90% by weight). While hydrogels are generally water‐insoluble, certain environmental factors, such as extreme pH or enzymatic degradation, can cause them to dissolve or degrade in water.^[^ ^132^, ^133^ ^]^ They can vary physical and chemical properties through the chemical synthesis process, where long molecular chains containing hydrophilic groups attach to each other. Traditional hydrogels have weak mechanical strength and lack intelligence. Recent advances in the field have led to the rise of intelligent hydrogels, also known as smart hydrogels, which can respond to changes in the environment, whether it be physical or chemical stimuli, in this case, thermal stimuli.^[^ ^59^, ^134^, ^135^ ^]^

Thermally responsive hydrogels undergo volume phase transitions—swelling or deswelling—in response to temperature changes. This behavior is governed by a temperature‐dependent shift in the balance between hydrophilic and hydrophobic interactions within the polymer network, which alters the hydrogel's solubility and can induce a sol‐gel phase transition from a flowing sol‐phase to a structured gel‐phase.^[^ ^59^ ^]^ Depending on the specific hydrogel, increasing temperature may lead to either swelling or shrinking, and these materials are typically classified as negatively thermo‐sensitive (NTS) or positively thermo‐sensitive (PTS). NTS hydrogels shrink upon heating, while PTS hydrogels swell. The transitions are driven by enthalpic and entropic changes in hydrogen bonding and hydrophobic interactions between polymer segments and water.^[^ ^59^, ^134^, ^136^ ^]^

For NTS hydrogels such as PNIPAAm, the material exhibits a lower critical solution temperature (LCST), above which it becomes hydrophobic and expels water. PNIPAAm is widely used due to its sharp LCST around physiological temperatures (≈32 °C), and its thermal behavior can be tuned through copolymerization. In contrast, PTS hydrogels like gelatin, agarose, and amylose exhibit an upper critical solution temperature (UCST), above which hydrophilic interactions dominate, leading to swelling. In both cases, water diffusion into or out of the hydrogel matrix drives the volume change.^[^ ^59^, ^137^ ^]^

This thermal response also alters the hydrogel's mechanical properties. Swollen hydrogels typically exhibit lower Young's moduli because the polymer chains are diluted by water, which acts as a plasticizer, increasing chain mobility and elasticity. Upon deswelling, the network becomes denser and stiffer, increasing Young's modulus but potentially reducing elasticity if deformation exceeds a brittle limit. These mechanical responses are also influenced by the polymer composition and crosslinking density, with higher crosslinking generally increasing stiffness and resistance to deformation.^[^ ^138^ ^]^ Additionally, environmental factors such as pH can significantly affect swelling behavior and actuation performance.^[^ ^134^, ^139^ ^]^

**Figure 5:**
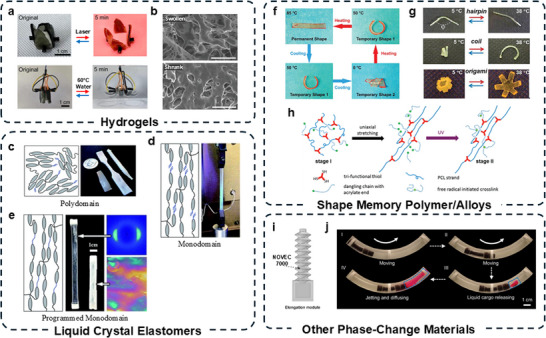
Thermally responsive materials for thermally actuated soft robots. a) Photographs of PNIPAM‐based hydrogels triggered by 808 nm laser or 60 °C water (Reproduced with permission.^[^ ^123^ ^]^ Copyright 2024, Elsevier). b) SEM image of SEM images of double network PNIPam hydrogel at a swollen state and shrunken state. Scale bar is 10 µm (Reproduced with permission.^[^ ^124^ ^]^ Copyright 2025, Wiley‐VCH). c, d, e) RM 257‐based LCE schematic illustrations of mesogens, spacers, crosslinkers, and experimental images. (c) Initial polydomain structure stage, post‐fabrication, pre‐stretch. (d) Temporary monodomain structure, post‐stretch along vertical axis, pre‐photo crosslink. (e) Permanent monodomain structure, post‐stretch, post‐photopolymerization under UV (c, d, and e: Reproduced with permission.^[^ ^125^ ^]^ Copyright 2015, Royal Society of Chemistry). f) Schematic illustration of the preparation of single‐phase two‐way shape actuator (Reproduced with permission.^[^ ^126^ ^]^ Copyright 2019, American Chemical Society). g) Multi‐shape memory stages of commercially available POEs (Reproduced with permission.^[^ ^127^ ^]^ Copyright 2014, American Chemical Society). h) Two‐way reversible shape memory demonstrated by end‐capped poly(octylene adipate) (Reproduced with permission.^[^ ^128^ ^]^ Copyright 2015, American Chemical Society). i) Schematic of focused ultrasound phase transition‐driven elongation actuator which would deform directionally via vaporization of NOVEC 7000. (j) Sequential thermal actuation and rapid liquid cargo release are induced by localized ultrasound (i and j: Reproduced with permission.^[^ ^129^ ^]^ Copyright 2024, Nature Publishing Group).

**Figure** **5**a demonstrates the biomimetic actuation behavior of a PNIPAM‐based actuator. The top panels show a bionic flower that gradually blooms under an 808 nm laser, driven by photothermal conversion through MXene‐enhanced anisotropic hydrogel layers. The bottom panel demonstrates an artificial octopus exhibiting rapid and multidirectional bending at 60 °C, enabled by the actuator's high tensile strength (94 kPa) and elongation (1014%).^[^ ^123^ ^]^ At the microscopic level, Figure 5b highlights the morphology of a double network hydrogel in its swollen and shrunken states. In the swollen state, a compact and robust polymer network is observed, while in the shrunken state, the polymer chains appear more agglomerated, with reduced pore sizes and visible phase separation due to bound water loss during shrinkage. The image reveals the crosslinked architecture and the inherent porosity of the hydrogel, including interconnected pores.^[^ ^124^ ^]^ Pore interconnectivity is a key factor in determining water diffusion rates within the hydrogel. Variations in pore size directly impact the hydrogel's thermal responsiveness, swelling/deswelling kinetics, and directional actuation behavior.^[^ ^140^ ^]^ Beyond porosity, molecular diffusivity within hydrogels influences actuation and is governed by factors such as the polymer network's mesh size, degree of crosslinking, and thermodynamic interactions between the network and permeating species. These parameters vary dynamically during the swelling or deswelling process.

#### 3.2.2 Liquid Crystal Elastomers

LCEs are smart materials composed of liquid crystal molecules (mesogens) and a polymer network. Their actuation arises from the collective alignment and reorientation of mesogenic units—rod‐like molecules embedded in the polymer matrix—which respond to external stimuli such as heat, light, or fields.^[^ ^141^, ^142^ ^]^ This molecular ordering gives rise to macroscopic, often anisotropic, shape changes. The hallmark of LCEs lies in their ability to undergo large, reversible deformations by exploiting a thermally driven phase transition between the nematic and isotropic states.

In the nematic phase, mesogens align along a common direction known as the director, establishing orientational order while remaining positionally disordered. As temperature increases, a transition to the isotropic phase occurs, disrupting mesogen alignment. This leads to contraction along the director axis and, depending on material constraints, may cause expansion or redistribution of strain in other directions. Upon cooling, the nematic order re‐establishes, allowing the material to recover its original shape (Figure 5c–e).

Two thermal parameters critically govern LCE actuation: the glass transition temperature (T_g_) and the nematic‐to‐isotropic transition temperature (T_ni_). T_g_ marks the onset of segmental mobility in the polymer network. Below T_g_, the material is glassy and rigid, preventing mesogen rearrangement and shape change. Above T_g_, the network becomes rubbery and elastic, enabling deformation in response to mesogen reorientation. For thermal actuation to occur, the actuation temperature must be above T_g_. T_ni_ determines the threshold for phase transition between the nematic phase and the isotropic phase. Careful tuning of the gap between T_g_ and T_ni_ is essential to achieving fast, reversible actuation.^[^ ^143^, ^144^ ^]^

Mesogen alignment within the polymer network significantly affects actuation efficiency. Major alignment strategies include mechanical alignment, surface‐enforced alignment, flow‐directed alignment, and field‐assisted alignment.^[^ ^145^ ^]^ Mechanical alignment applies uniaxial strain during partial polymerization to orient mesogens, with subsequent crosslinking fixing the alignment. It is simple and effective for large volumes but limited in patterning precision. Surface‐enforced alignment uses treated layers to anchor mesogens at the surface, promoting uniform alignment into the bulk, though it requires specialized substrates and is sensitive to surface quality.^[^ ^146^ ^]^ Flow‐directed alignment leverages shear in confined flows (e.g., microchannels) to align mesogens, making it suitable for fibers and films, but it requires precise flow control.^[^ ^147^ ^]^ Field‐assisted alignment uses electric or magnetic fields to orient mesogens based on their anisotropy, offering reconfigurable alignment, but it requires costly equipment and becomes less effective for thick or highly crosslinked samples.^[^ ^148^, ^149^ ^]^

Finally, the thermomechanical performance of LCE is closely linked to its reversible strain, a critical metric that quantifies actuation efficiency. This parameter highly depends on the fixity of the LCE polymer, which is dependent on the molecular weight, the cross‐linking reaction used, and alignment methods. Fixity is calculated by $Fixity\;\left(\%\right)=\frac{\varepsilon_{fixed}}{\varepsilon_{appiled}}$, where ɛ_*appiled*_ is the programming strain before photo‐crosslinking and ɛ_*fixed*_ is the amount of permanent strain after photo‐crosslinking.^[^ ^150^ ^]^

LCEs are unique in that their actuation is tightly coupled to molecular order and phase behavior. The thermally induced phase change alters both mechanical and optical properties, such as birefringence and opacity, making them valuable in soft robotics, tunable optics, and artificial muscles. Their performance depends not only on the presence of liquid crystalline phases but also on how well the order can be programmed, retained, and manipulated under operational conditions.

#### 3.2.3 Shape‐Memory Alloys/Polymers

SMAs are metallic materials capable of “remembering” and returning to their original shape when exposed to thermal stimuli. Their high force‐to‐weight ratio and energy density make them appealing for soft robotics.^[^ ^151^ ^]^ Notably, nickel‐titanium alloy exhibits remarkable elasticity and super elasticity within a specific temperature range, transitioning from the martensitic phase to the austenitic phase. However, their inherent rigidity restricts their application in soft robotics.^[^ ^152^ ^]^

SMPs, in contrast, are a class of smart materials capable of deforming then returning to their original shape in response to stimuli. Compared to SMAs, SMPs offer lower density, greater flexibility, and the potential for more efficient, lightweight, and portable soft robotics systems. In recent years, significant progress has been made in developing SMPs with diverse network topologies, surface structures, and porosities, along with reversible properties and responsiveness to various stimuli such as heat, light, and pH.^[^ ^153^ ^]^

Similar to LCEs, the T_g_ is crucial for the deformation of SMPs as it marks the switch between a rigid, glassy state and a flexible, rubbery state. Below T_g_, SMPs can hold a temporary shape; reheating above T_g_ allows them to recover their original form.^[^ ^154^ ^]^ This reversible thermal behavior enables their shape memory effect. In contrast, SMAs rely on a martensitic‐austenitic phase transformation, not T_g_. While SMP actuation depends on polymer chain mobility across T_g_, SMAs actuate through a solid‐state crystal structure change. Both mechanisms are thermally driven but stem from different material physics.

A distinctive feature of SMPs is their programmability; SMP shapeshifting can be manipulated externally post‐fabrication. SMPs can be broadly classified into three types based on their shape‐memory behavior: one‐way SMPs (1W‐SMPs), multi‐shape memory polymers (multi‐SMPs), and two‐way SMPs (2W‐SMPs). As the simplest class, 1W‐SMPs are programmed to fix one temporary shape and recover their permanent shape upon stimulation (known as the one‐way dual‐shape memory effect).^[^ ^155^ ^]^ While 1W‐SMP thermal actuation can be found in some applications, they do not represent true multi‐shape memory behavior inherent to advanced SMPs such as multi‐SMPs or 2W‐SMPs. In the early 2000s, more complex SMPs, multi‐SMPs, were developed. Multi‐SMPs can remember and recover multiple temporary shapes in addition to their original, permanent shape.^[^ ^58^, ^155^, ^156^ ^]^ However, multi‐SMPs do not necessarily mean that the shape change is reversible. Reversible SMPs are classified as 2W‐SMPs and can reversibly switch between two or more distinct programmed shapes, enabling continuous, controlled, and reversible shape changes highly desirable in soft robotics.

For example, Figure 7f shows the process of synthesizing a 2W‐SMP based on a partially cross‐linked, semicrystalline poly(ɛ‐caprolactone) network.^[^ ^126^ ^]^ It is important to note that the dual‐cure process stabilizes two states, which allows for full reversibility. Figure 7g shows a 2W‐SMP transitioning from temporary shape 1 (circular at 50 °C) to temporary shape 2 (coiling at 0 °C), reverting to its permanent shape when heated to 85 °C.^[^ ^127^ ^]^ Figure 7h demonstrates a two‐way reversible shape memory of three different trained shapes: hairpin, coil, and Origami structure.^[^ ^128^ ^]^

SMPs generally have higher mechanical strength compared to hydrogels and LCEs, withstanding higher stresses and being more durable. Additionally, they offer high shape‐memory capabilities, allowing for multi‐step complex deformations.^[^ ^157^ ^]^ However, SMPs are limited to specific temperature ranges for activation, with slower response times in comparison to LCEs, and may require precise environmental control for specific activation.

#### 3.2.4 Phase‐Change Fluids

A mechanism increasingly employed in thermal actuators relies on phase‐change‐driven fluidic systems, where the volumetric expansion of a fluid–typically through liquid‐to‐gas phase transition–is harnessed for actuation. These systems exploit the thermodynamic response of fluids to temperature changes rather than introducing a new class of smart materials. Actuation is achieved by heating sealed fluid to or beyond its boiling point, generating internal pressure and volume changes that drive mechanical motion.

One notable example is the electroconductive fiber‐reinforced phase transition actuator (E‐FPTA), which uses a low‐boiling‐point fluid, NOVEC 7100, in a silicone rubber elastomer sleeve embedded with flexible metallic fibers that act as both Joule heaters and structural guides.^[^ ^158^ ^]^ The E‐PFTA demonstrates multiple form factors by customizing the metallic fiber pattern, including extending, contracting, twisting, and bending. The extending‐type E‐PTFA achieves over 100% strain and Newton‐level force output with under 17 W of power, making it ideal for untethered, portable use powered by batteries.

Like the use of NOVEC 7100 in the E‐FPTA, other thermally responsive actuators utilize similar fluids such as NOVEC 7000,^[^ ^159^, ^160^ ^]^ ethanol,^[^ ^161^ ^]^ water,^[^ ^162^ ^]^ etc. These fluids are typically encapsulated in an elastic chamber, and a controlled amount of heat is applied through an integrated heater, electromagnetic waves, or other external stimuli, enabling precise regulation of internal pressure and volume. Figure 7i illustrates the focused ultrasound phase transition‐driven elongation actuator, which undergoes directional deformation when NOVEC 7000 reaches its boiling point.^[^ ^129^ ^]^ This enables functions such as targeted drug delivery or untethered robotic movement. Figure 7j shows the thermal actuation process where localized ultrasound triggers sequential locomotion (stages I and II), then triggers rapid liquid cargo release (stages III and IV). The vaporization of NOVEC 7000 generates internal pressure, driving the rapid controlled expulsion of contents. This mechanism enables reversible, low‐temperature actuation, making it suitable for untethered soft robotic applications.

## 4 Structural Designs

The motion of thermal soft actuators is determined by the interplay between microscale material deformation and macroscale structural design, often involving multiple materials. These actuators can be classified by the dimension of their deformation. 1D actuators exhibit linear movements such as stretching or contraction, as seen in artificial muscles and tendons. 2D deformation involves planar bending or folding, typically found in multilayer, sheet‐based structures. More complex designs enable 3D shape morphing, including twisting, spiral, multi‐axis bending, and braid‐like configurations. These diverse modes of deformation contribute to the versatility and functional complexity of thermal soft actuators. In this section, we highlight representative examples of 1D, 2D, and 3D deformation modes, along with their fundamental characteristics and applications.

### 4.1 1D Extension/Contraction

One of the most direct methods of converting thermal energy into mechanical deformation is through thermal expansion or contraction in response to heat. Actuators with fiber or tube‐like geometries that can extend or contract are particularly promising for artificial muscle applications. For instance, Figure 3a illustrates an electrothermal actuator composed of a hollow LCE fiber, where liquid metal is used as an internal heating element.^[^ ^163^ ^]^ These hollow LCE fibers present significant potential for innovative design and fabrication in areas such as soft robotics, adaptive structures, smart wearables, and optical systems. Thermal actuators operating in extension/contraction mode offer key advantages over bending or twisting actuators, particularly in applications requiring linear motion. They can generate higher axial forces due to the full use of their cross‐section, provide more efficient and direct force transmission, and are easier to integrate into systems where motion is aligned with the load direction. While bending and twisting modes are useful for complex deformations and shape morphing, extension/contraction actuators are generally better suited for tasks that demand strong, directional actuation with simpler mechanical design.

### 4.2 2D Bending/Folding Structure

2D bending and folding structures are essentially bimorph structures, consisting of two materials bonded together, typically with different coefficients of thermal expansion (CTE). As the temperature changes, the materials expand at different strains, resulting in bending or twisting deformation. The curvature of a bimorph can be calculated with Timoshenko's equation:^[^ ^173^ ^]^

(1) $$k=\frac{6\left(\alpha_{1}-\alpha_{2}\right)\left(T-T_{0}\right){\left(1+m\right)}^{2}}{h\left[3{\left(1+m\right)}^{2}+\left(1+mn\right)\left(m^{2}+\frac{1}{mn}\right)\right]}$$

where *m = t*_1_ */t*_2_ with *t*_1_ and *t*_2_ as the thicknesses of the two layers, *h = t*_1_ *+ t*_2_, *n = E*_1_ */E*_2_ with *E*_1_ and *E*_2_ as the Young's modulus of the two layers, *T*_0_ is the initial temperature of the actuator, *T* is the current temperature, and *α*_1_ and *α*_2_ are the CTEs of the two layers.

**Figure 6:**
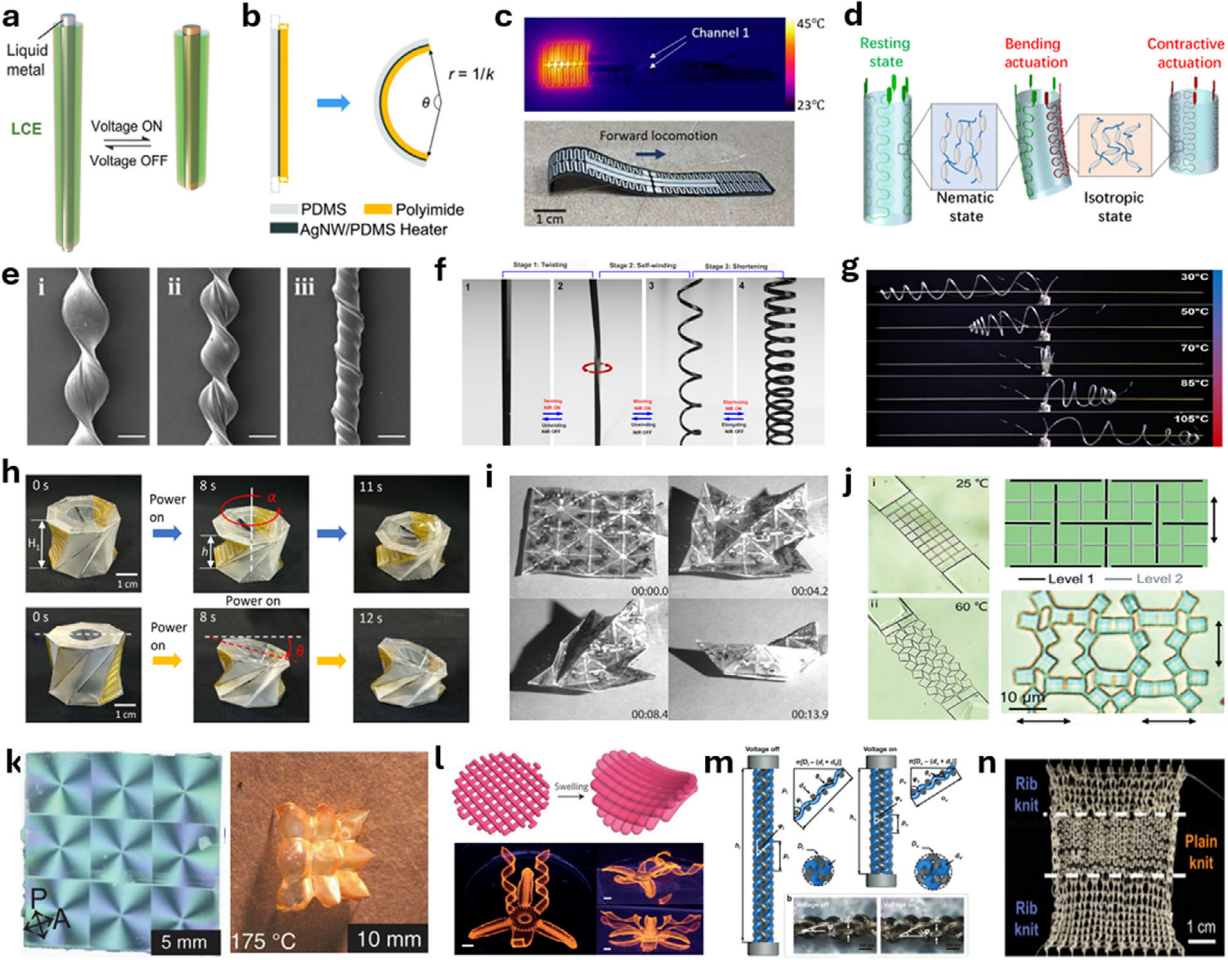
Structural designs of soft thermal actuators. a) An electrothermal actuator enabled by a hollow LCE fiber filled with liquid metal (Reproduced with permission.^[^ ^163^ ^]^ Copyright 2024, Wiley‐VCH). b) A bimorph actuator with AgNW/ PDMS heater sandwiched between PDMS and PI (Reproduced with permission.^[^ ^17^ ^]^ Copyright 2017, Royal Society of Chemistry). c) A Soft crawling robot with distributed patterned heater to control its locomotion direction (Reproduced with permission.^[^ ^20^ ^]^ Copyright 2023, American Association for the Advancement of Science). d) Tubular LCE actuator with three serpentine shaped metallic wire heaters (Reproduced with permission.^[^ ^18^ ^]^ Copyright 2019, American Association for the Advancement of Science). e) LCE artificial tendril with asymmetric core‐sheath structure (Reproduced with permission.^[^ ^164^ ^]^ Copyright 2023, Wiley‐VCH). Scale bars: 200 µm. f) A phototunable self‐oscillating system powered by a self‐winding fiber actuator (Reproduced with permission.^[^ ^165^ ^]^ Copyright 2021, Nature Publishing Group). g) A fiber‐shaped artificial nylon muscles with spiral design (Reproduced with permission.^[^ ^166^ ^]^ Copyright 2016, PNAS). h) A modular Origami robot with integrated thermal actuators showing the contraction and bending motions (Reproduced with permission.^[^ ^34^ ^]^ Copyright 2024, PNAS). i) A self‐folding 32‐tile self‐folding Origami sheet transforming into a boat (Reproduced with permission.^[^ ^167^ ^]^ Copyright 2010, PNAS). j) Long‐strip kirigami LCE structure with 2 × 4 units (32 hinged squares) and its configuration snapshots at different temperatures (Reproduced with permission.^[^ ^168^ ^]^ Copyright 2021, Wiley‐VCH). k) A periodically programmed LCE film with nine cones arisen (Reproduced with permission.^[^ ^169^ ^]^ Copyright 2015, American Association for the Advancement of Science). l) Schematic and photograph of the direct ink writing enabled bi‐layer hydrogel architectures (Reproduced with permission.^[^ ^170^ ^]^ Copyright 2016, Nature Publishing Group). m) Illustration and microscope images of a braided thermal actuator (Reproduced with permission.^[^ ^171^ ^]^ Copyright 2023, Wiley‐VCH). n) A knitted LCE actuator with distributed knitting patterns (Reproduced with permission.^[^ ^172^ ^]^ Copyright 2023, Wiley‐VCH).

**Figure** **6**b,c shows two examples of a bending thermal actuator/robot.^[^ ^17^, ^20^ ^]^ By leveraging the large CTE difference between polydimethylsiloxane (PDMS) and PI and the effective Joule heating enabled by the AgNW network, a soft actuator showed 720°bending angle within 30 s.^[^ ^17^ ^]^ Other than the global bending, researchers have explored distributed local bending by patterning the conductive material and controlling the temperature distributions, offering excellent programmability.^[^ ^20^ ^]^ Bimorph structures can be designed to fulfill various tasks, such as gripping or navigating through complex environments. In addition to the bilayer structure, a similar approach is to use the deformation mismatch in different parts of the structure to trigger bending in the overall structure. Figure 6d shows a cylindrical LCE tube with embedded metallic wire heaters, which demonstrated omnidirectional bending by selective heating of different embedded wires (and hence local shrinkage of different parts of the LCE tube).^[^ ^18^ ^]^ In this tubular structure, heating the LCE/heater composite induces localized uniaxial contraction. However, because the three actuators are evenly distributed around the tube, activating a single actuator produces an overall bending motion due to the asymmetric contraction. The combination of large deformation and unparalleled programmability showcases the significant potential of bimorph‐based actuators in advancing soft robotics technologies.

### 4.3 3D Twisting/Spiral Structure

Bending and twisting/spiral deformation in thermal actuators share the common mechanism of differential thermal expansion and are both widely used in soft robotic systems. While bending typically involves a fixed bending axis and produces smooth, uniform out‐of‐plane curvature, twisting and spiral deformations result from a rotating or distributed bending axis, leading to non‐uniform, often periodic, deformation patterns such as torsion or helical coiling. The former is generally simpler to control and predict, whereas the latter enables more complex motions but often requires careful structural design to induce the desired torsion.

Twisting and spiral structures in thermally actuated soft robots often draw inspiration from nature, e.g., tendrils, vines, and snakes, where the robot can undergo significant deformation through rotation or twisting motions.^[^ ^164^, ^165^, ^166^, ^174^ ^]^ These structures often employ helical or spiral geometries that enhance displacement and play a crucial role in mimicking biological motions.

When subjected to thermal stimuli, the materials comprising these spirals change dimensions, inducing a twisting effect that can be harnessed for various applications, including locomotion and manipulation tasks. For example, an LCE artificial tendril with asymmetric core‐sheath structure design showed the ability to perform complex hyponastic movements in response to environmental temperature stimuli^[^ ^164^ ^]^ (Figure 6d). Figure 6e shows a phototunable self‐oscillating system powered by a self‐winding fiber actuator, leveraging light‐induced actuation to generate diverse oscillation modes.^[^ ^165^ ^]^ The system exhibited robust, autonomous motion and demonstrated potential applications in intelligent machines and renewable energy harvesting. Figure 6f shows a fiber‐shaped artificial muscle with a spiral design, capable of stretching over 1000% of its original size while lifting substantial weights, thus emulating biological muscle properties.^[^ ^166^ ^]^ These innovative designs illustrate how twisting and spiral structures can be used to enhance robotics and biomedical devices, enabling softer, safer, and more efficient mechanical systems.

### 4.4 Other 3D Deformable Structures

3D deformable structures, encompassing Origami and Kirigami designs, dome and saddle shapes, and knitted structures, to name a few, represent an important class in the design of thermally actuated soft robots. Origami and Kirigami utilize folds and cuts in materials, transforming flat sheets into complex 3D shapes.^[^ ^175^ ^]^ Figure 6g shows an origami‐structured actuation unit that can be assembled and programmed for multi‐model locomotion (forward and backward, steering).^[^ ^34^ ^]^ The Origami unit was equipped with two bimorph thermal actuators that can achieve localized sharp folding. In Figure 6h, a reprogrammable Origami sheet crafted from interconnected triangular sections has demonstrated the capability of shapeshifting from flat to a boat and an airplane.^[^ ^167^ ^]^ A reconfigurable microscale Kirigami structure made from LCEs offered unique advantages in terms of flexibility and responsiveness to external stimuli.^[^ ^168^ ^]^ These Origami/Kirigami structures with appropriate mechanical designs can significantly enhance performances in various applications, particularly in developing smart biological movements and programmable/reprogrammable transformation.

Other than Origami/Kirigami structures, dome and saddle structures that exploit changes in Gaussian curvature are also widely used in thermally actuated soft robots to generate unique shape morphing patterns. Figure 6j shows spatially patterned LCE flat sheets that can transform into 3D dome/cone groups through controlled 3D bending.^[^ ^169^ ^]^ The programmable mechanical response of these materials offers great potential for fabricating substrates for flexible devices in aerospace, medicine, or consumer goods. Inspired by botanical systems, a printed composite hydrogel architecture showed localized, anisotropic swelling, which can generate complicated 3D deformations that can change with time^[^ ^170^ ^]^ (Figure 6k).

Additionally, as shown in Figure 6l,m, braided and knitted structures can bring a unique combination of flexibility and strength, enabling thermally actuated soft robots to respond to temperature changes with enhanced load‐bearing capabilities.^[^ ^171^, ^172^ ^]^

By employing advanced materials alongside these structural designs, researchers can develop soft robots capable of executing intricate tasks, from soft gripping, crawling to dynamic biomedical applications. The synergy of thermally actuated mechanisms and 3D deformable shapes significantly enriches the functionality and potential use cases of soft robotics in real‐world applications.

### 4.5 Instability

The materials selection and structural designs discussed above have led to significant advancements in soft thermal actuators and robotics, enhancing both functionality and applicability. However, owing to the intrinsic low thermal conductivity of most soft materials, a major limitation of such thermally actuated soft robots is the relatively low speed. Increasing the speed for soft robots is a general challenge in the field, especially for entirely soft robots (without the assistance of rigid skeletons, frames, etc).^[^ ^22^, ^176^, ^177^ ^]^

**Figure 7:**
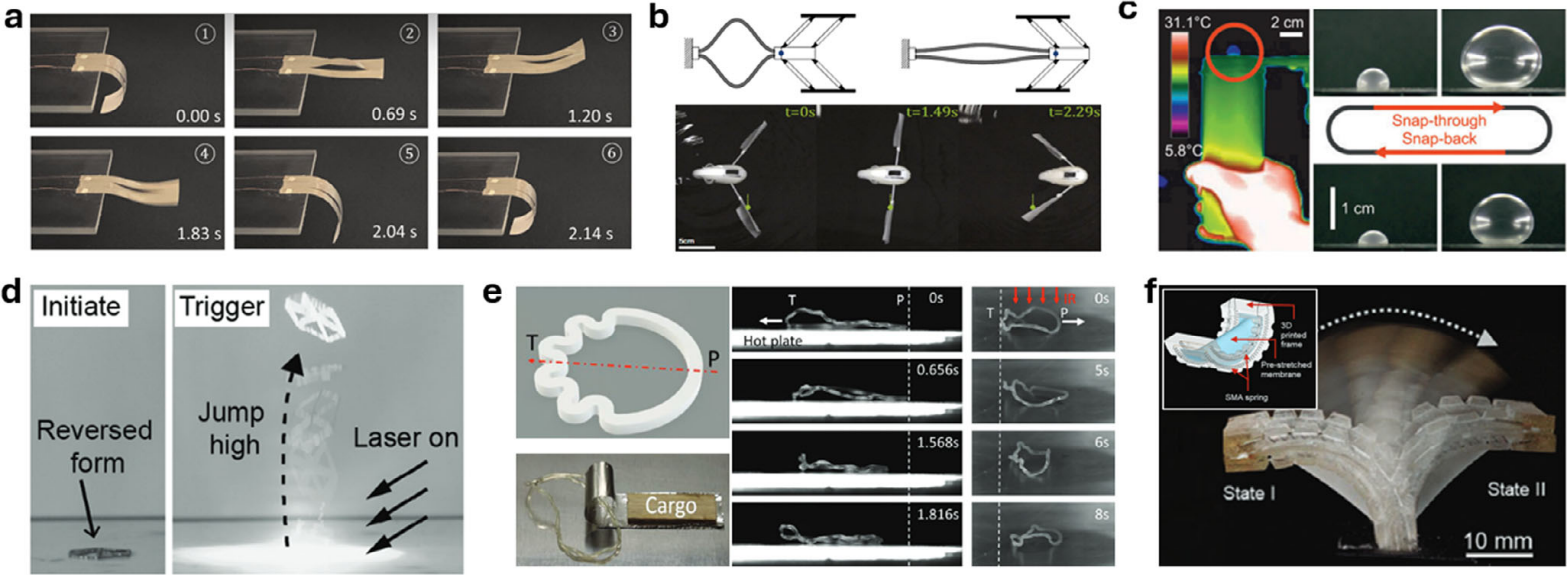
Instability design in soft thermal actuators. a) Screenshots of a three‐ribbon‐structured soft bimorph actuator with snap‐through instability (Reproduced with permission.^[^ ^19^ ^]^ Copyright 2022, Mary Ann Liebert). b) Schematics and photographs of an untethered soft swimming robot with SMP muscles and a group of bistable beam elements (Reproduced with permission.^[^ ^178^ ^]^ Copyright 2018, National Academy of Sciences). c) a high‐speed body temperature‐triggered soft ballon robot with mechanical instability (Reproduced with permission. (Reproduced with permission.^[^ ^179^ ^]^ Copyright 2022, Mary Ann Liebert). d) A 4D printed metal jumper inspired by biological insect jumping mechanisms (Reproduced with permission.^[^ ^180^ ^]^ Copyright 2024, Wiley‐VCH). e) A self‐sustained snapping ring structure with locomotion and load carrying capabilities (Reproduced with permission.^[^ ^181^ ^]^ Copyright 2023, Wiley‐VCH). f) A highly dynamic bistable soft actuator composed of a prestretched membrane sandwiched between two 3D printed frames with embedded SMA coils (Reproduced with permission.^[^ ^182^ ^]^ Copyright 2023, Wiley‐VCH).

Mechanical instability offers a novel approach to enhancing the speed of thermally actuated soft robots. A bimorph thermal actuator with AgNW/PDMS composite film as the heating material exploited snap‐through instability to significantly increase the actuation speed.^[^ ^19^ ^]^ The actuator yielded a bending speed as high as 28.7 cm^−1^ s^−1^, 10 times that without the snap‐through instability (**Figure** **7**a). Figure 7b shows an untethered, soft swimming robot, which can complete preprogrammed tasks with shape memory polymer (SMP) muscles and a group of bistable beam elements.^[^ ^178^ ^]^ With the stimuli of environmental temperature, the robot achieved multiple functions, including moving forward, turning, reversing, and/or delivering a cargo. Figure 7c shows a mechanism for high‐speed movements in soft robots using body temperature‐triggered mechanical instabilities. The design leveraged the unique properties of a balloon that responds to thermal stimuli and brings about a liquid/gas phase transition in a suitable volatile fluid. This work demonstrated large deformations up to 300% area expansion within a few milliseconds.^[^ ^179^ ^]^ Researchers also developed 4D printed metal jumpers inspired by biological insect jumping mechanisms (Figure 7d). The design incorporated snapping as the key instability, enabling rapid propulsion through mechanical energy release. The SMA net‐shell achieved a jump height of 60 body lengths (BL) and takeoff speed up to 300 BL s^−1^.^[^ ^180^ ^]^ In Figure 7e, a self‐sustained snapping structure has been utilized to drive motion and carry loads. The design of free‐standing wavy rings allowed them to autonomously dance and perform complex movements using the controlled instability of snapping.^[^ ^181^ ^]^ Figure 7f shows a highly dynamic, bistable soft actuator that facilitated multimodal locomotion in soft robots. These actuators were composed of a prestretched membrane sandwiched between two 3D printed frames with embedded SMA coils that generate a force of 0.3 N after 0.2 s of electrical activation.^[^ ^182^ ^]^

The instability designs take advantage of snap‐through (and sometimes snap‐back) behavior to drive movement, leading to enhanced speed and agility. By integrating these capabilities into thermally actuated soft robots, it is promising to create highly functional and adaptable soft robotic systems.

## 5 Applications

Soft robots represent a significant advancement in the field of robotics, leveraging the unique properties of soft materials to enable a broad spectrum of applications, such as environmental exploration, gripping and manipulation, biomedical applications, and human interaction and aesthetic applications. Here are representative works in these applications using thermally actuated soft robots.

### 5.1 Locomotive Soft Robots

This section discusses the deployment of thermally actuated soft robots in unpredictable or extreme environments for exploration, inspection, or interaction with natural ecosystems. The largest class of robots that fall under this category is locomotive soft robots.

**Figure 8:**
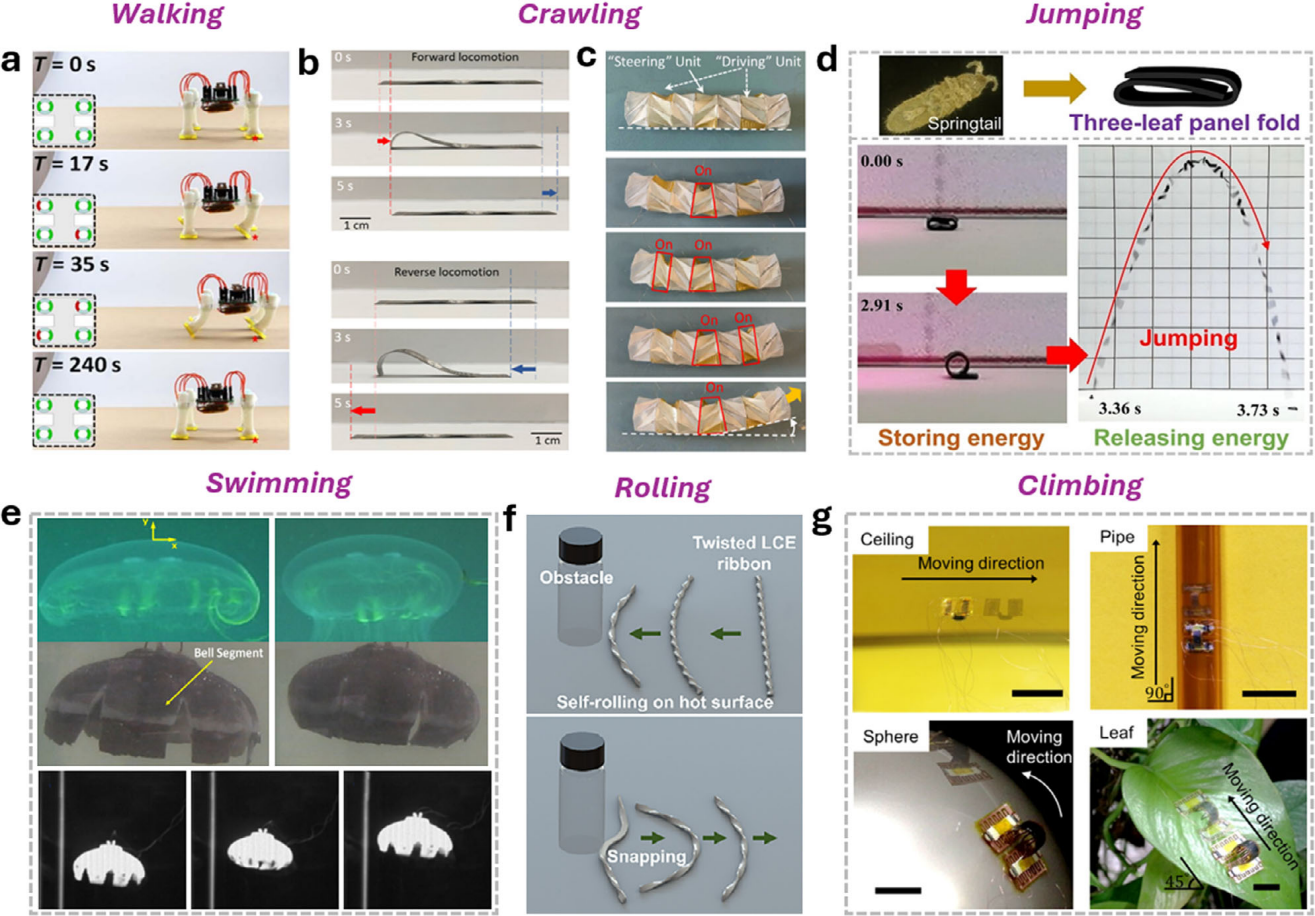
Thermally actuated soft locomotion robots. a) An untethered multi‐gait walking robot enabled by tubular thermal actuators (Reproduced with permission.^[^ ^18^ ^]^ Copyright 2019, American Association for the Advancement of Science). b) A caterpillar inspired soft crawling robot with distributed and programmable heaters (Reproduced with permission.^[^ ^20^ ^]^ Copyright 2023, American Association for the Advancement of Science). c) Snapshots of a Kresling Origami structured modular crawling robot in the steering motion (Reproduced with permission.^[^ ^34^ ^]^ Copyright 2024, National Academy of Sciences). d) A light‐driven soft jumping robot based on a double‐folded LCE ribbon actuator with a monolithic three‐leaf panel fold structure (Reproduced with permission.^[^ ^194^ ^]^ Copyright 2023, Wiley‐VCH). e) A jellyfish‐like swimming robot actuated by SMA composite actuators (Reproduced with permission.^[^ ^186^ ^]^ Copyright 2011, IOP Publishing). f) A self‐rolling robot showing the snapping and reversed locomotion after encountering obstacles (Reproduced with permission.^[^ ^196^ ^]^ Copyright 2022, National Academy of Sciences). g) A climbing robot demonstrating locomotion on a glass ceiling, a PI cylinder (diameter: ≈10 mm), a glass sphere (diameter: ≈25 cm), and leaf of *Epipremnum aureum* (Reproduced with permission.^[^ ^190^ ^]^ Copyright 2022, National Academy of Sciences). The scale bar is 10 mm.

Thermally actuated soft locomotion robots utilize thermal energy to create adaptable and flexible machines capable of navigating complicated environments. These robots can exhibit various modes of movement, including walking,^[^ ^18^ ^]^ crawling,^[^ ^19^, ^20^, ^34^, ^112^, ^183^, ^184^, ^185^, ^189^, ^192^ ^]^ jumping,^[^ ^112^, ^194^ ^]^ rolling,^[^ ^195^, ^196^ ^]^ swimming,^[^ ^186^, ^187^ ^]^ and climbing^[^ ^190^ ^]^ (**Figure** **8**a–f). Such a wide versatility makes them ideal for applications requiring mobility across challenging terrains.

**Table** **1** shows a comparison between different locomotion robots in terms of heating mechanisms, materials, and key performances. Crawling motion is the most widely studied among different locomotion modes. It is important to note that there are two primary types of crawling. The first is inching, similar to the movement of an inchworm, where the middle of the body curves upward, and the head or tail moves closer to or farther from the opposite end. The second is peristaltic crawling, commonly observed in earthworms, caterpillars, and even snakes, which involves the sequential contraction and relaxation of body segments. For thermally actuated crawling robots, speed is a critical challenge for thermally actuated soft robots because of the relatively low thermal conductivity of soft materials. For example, an LCE/PI bimorph actuator showed a crawling speed of 0.032 mm s^−1^ or 0.0011 BL s^−1^. This speed is significantly slower than typical pneumatic, dielectric, and magnetic crawling robots.^[^ ^184^, ^197^, ^198^, ^199^ ^]^ To address this issue, researchers have adopted mechanical instability designs for thermal actuators to enhance the speed, as discussed in Section 4.5. The snap‐through instability significantly increases the actuation speed, up to 5 mm s^−1^ (1.04 BL s^−1^).^[^ ^19^ ^]^ Compared to crawling and rolling soft robots, jumping and swimming robots generally show higher speed because the resistance/friction from the environment (air, water) is generally smaller than that for locomotion on the ground. For example, a 4D printed rolling robot demonstrated a fast speed of 8 mm s^−1^ when placed on a hot plate.^[^ ^195^ ^]^ A springtail‐inspired soft robot with a three‐leaf panel fold structure delivered excellent jump performance, with the highest jumping height, longest jumping distance, and maximum take‐off velocity reaching 87 BL, 65 BL, and 930 BL s^−1^, respectively^[^ ^194^ ^]^ (Figure 8d). A jellyfish‐inspired swimming robot can swim at the peak speed of 54.5 mm s^−1[^ ^186^ ^]^ (Figure 8e).

**Table 1:** Thermally actuated soft locomotion robots.

	Heating mechanism	Heat source	Thermally responsive materials	Heater programmability	Locomotion modes	Locomotion speed	Cooling speed (°C s^−1^)	CoT
[183]	Joule heating	Ni‐Cr wires	LCE/PI	×	Crawling (1‐directional)	0.73 mm s^−1^	3.2	5.77E + 06
[184]	Joule heating	Cr‐Au mesh	LCE/PI	√	Crawling (2‐directional)	0.031 mm s^−1^ (0.0011 BL s^−1^)	n/a	7.36E + 05
[18]	Joule heating	Cu wires	LCE	√	Walking (multi‐gait)	0.056 mm s^−1^ (0.0007 BL s^−1^)	0.85	5.00E + 04
[185]	Joule heating	SMA	SMA	×	Crawling (1‐directional)	2 mm s^−1^	n/a	n/a
[186]	Joule heating	SMA	SMA	×	Swimming	54.5 mm s^−1^	n/a	n/a
[187]	Joule heating	Cu	SMP	×	Swimming	28.8 mm s^−1^	n/a	n/a
[188]	Joule heating	Cu	PEG‐PU/PI	√	Crawling (2‐directional)	0.21 mm s^−1^ (0.0016 BL s^−1^)	n/a	5.00E +05
[189]	Joule heating	CNT	Nylon/CNT	×	Crawling (1‐directional)	0.55 mm s^−1^ (0.0072 BL s^−1^)	2.66	1.20E + 06
[190]	Joule heating	LIG	LCE	×	C limbing	0.018 BL s^−1^	n/a	2E4 (vertical) & 9E4 (horizontal)
[19]	Joule heating	AgNWs	PDMS	√	Crawling (1‐directional)	5 mm s^−1^ (1.05 BL s^−1^)	3.16	n/a
[34]	Joule heating	AgNWs	LCE/PI	√	Crawling (2‐directional & steering)	0.195 mm s^−1^	1.4	6.65E + 04
[20]	Joule heating	AgNWs	LCE/PDMS	√	Crawling (2‐directional & bioinspired body profile control)	0.72 mm s^−1^ (0.012 BL s^−1^)	0.416	4.88E + 04
[36]	EMI	SMA	SMA	×	Crawling (1‐directional)	0.1 mm s^−1^ (0.0025 BL s^−1^)	0.8	n/a
[52]	EMI	LM	LCE	√	Crawling (1‐directional)	0.0083 BL s^−1^	n/a	n/a
[112]	EMR	UV light	LCE	×	Crawling (1‐directional) & jumping	0.7 mm s^−1^ (0.0083 BL s^−1^)	n/a	n/a
[191]	EMR	UV light	LCE	√	Crawling (2‐directional)	0.24 mm s^−1^ (0.0165 BL s^−1^)	n/a	3.30E + 08
[192]	EMR	NIR light	GO	×	Crawling (1‐directional)	0.338 mm s^−1^ (0.0113 BL s^−1^)	n/a	n/a
[193]	EMR	NIR light	LCE/imNi8(4)	√	Crawling (1‐directional & steering)	0.33 mm s^−1^	5.45	n/a
[194]	EMR	NIR light	LCE	×	Jumping	2 m ^−1^s (initial speed)	n/a	n/a
[195]	Fluid Convection	Environment (hot plate)	LCE	×	Rolling	8 mm s^−1^	n/a	n/a
[196]	Fluid Convection	Environment (hot plate)	LCE	×	Rolling (obstacle avoiding)	0.4 mm s^−1^	n/a	n/a

Thermally actuated systems offer unique advantages for underwater or swimming robots, particularly due to the enhanced heat transfer properties of aquatic environments. The surrounding fluid facilitates rapid cooling, which can improve actuation speed and enable higher cycling frequencies compared to operation in air. This makes thermal actuators attractive for soft and flexible robotic swimmers that aim to mimic natural locomotion in a compact and noise‐free manner. An EMI actuated underwater crawling robot demonstrated effective heating and rapid deformation because the surrounding water enabled fast cooling of the bilayer actuator, supporting high‐frequency, continuous motion.^[^ ^52^ ^]^ However, several challenges must be addressed for practical deployment. Notably, while water enhances cooling, it can also lead to inefficient heating due to continuous heat loss, making heat accumulation and precise thermal control more difficult. This often necessitates higher energy input, which raises concerns about power consumption and thermal management, especially in untethered or battery‐powered systems. Furthermore, the integration of waterproof thermal insulation and the need for robust encapsulation to protect thermal elements from the environment add complexity to the design.

Other than the listed locomotion types, flying robots remain challenging for thermal actuation. A flier robot inspired by wind‐dispersed seeds, implemented using SMP, has been investigated, with a focus on its aerodynamic interactions during free fall.^[^ ^200^ ^]^

Another challenge for soft robots is to achieve bidirectional and programmable locomotion. As listed in Table 1, most soft thermal robots can only achieve one‐directional movement. However, for practical applications, soft robots require more sophisticated motion controls to effectively navigate complex terrains and perform specific tasks. Figure 8b shows a bioinspired crawling robot that mimics the inching locomotion of caterpillars, controlled by Joule heating of a patterned soft heater consisting of AgNW networks in an LCE‐based thermal bimorph actuator.^[^ ^20^ ^]^ With distributed heating patterns and switchable conducting channels, different temperature and hence curvature distributions were achieved, enabling bidirectional locomotion as a result of the friction competition between the front and rear end with the ground.

The third challenge is the steering motion of soft robots. The inching motion and peristaltic crawling motion of caterpillars have been widely studied for the design of soft robots, while the steering motion with local bending control remains challenging. To address this challenge, Wu et al. reported a modular soft Kresling Origami robot with peristaltic crawling motion (Figure 8c). A multi‐unit caterpillar‐inspired soft robot composed of active units and passive units is developed for steering locomotion, in addition to bidirectional locomotion, with precise bending curvature control of each active unit.^[^ ^34^ ^]^ To navigate through specific environments, such as obstacles or inclined/vertical surfaces, special functions have been explored for thermal locomotion robots. A twisted LCE ribbon can snap and bounce away from an obstacle.^[^ ^196^ ^]^ A micro‐bot equipped with electroadhesive pads can climb and move on a glass ceiling, a PI cylinder, a glass sphere, and natural leaves.^[^ ^190^ ^]^

Energy efficiency in thermal actuation is another important challenge, primarily due to the heat losses that occur to the surrounding environment. Cost of transport (CoT) = E/(mgd), as defined,^[^ ^20^, ^201^ ^]^ can be used to characterize the energy efficiency, where E is the input energy, m is the mass, g is the gravitational constant, and d is the moved distance. From the CoT listed in Table 1, it is observed that the robots with bioinspired locomotion patterns (e.g., multi‐gait walking,^[^ ^18^ ^]^ caterpillar body profile,^[^ ^20^ ^]^ multi‐segment peristalsis^[^ ^34^ ^]^) have CoTs that are an order of magnitude lower than the rest, indicating much higher energy efficiency. This suggests that bioinspiration is the key to energy efficiency in soft robots for locomotion.

### 5.2 Gripping and Manipulation

#### 5.2.1 Thermally Actuated Soft Grippers

Thermally actuated soft grippers showcase the advancement in robotic handling technology, particularly in applications where delicate interactions with objects are essential. These grippers adapt their shape and gripping force to accommodate an array of items with different stiffness, making them ideal for industries such as food production, healthcare, and manufacturing.^[^ ^215^ ^]^

**Figure 9:**
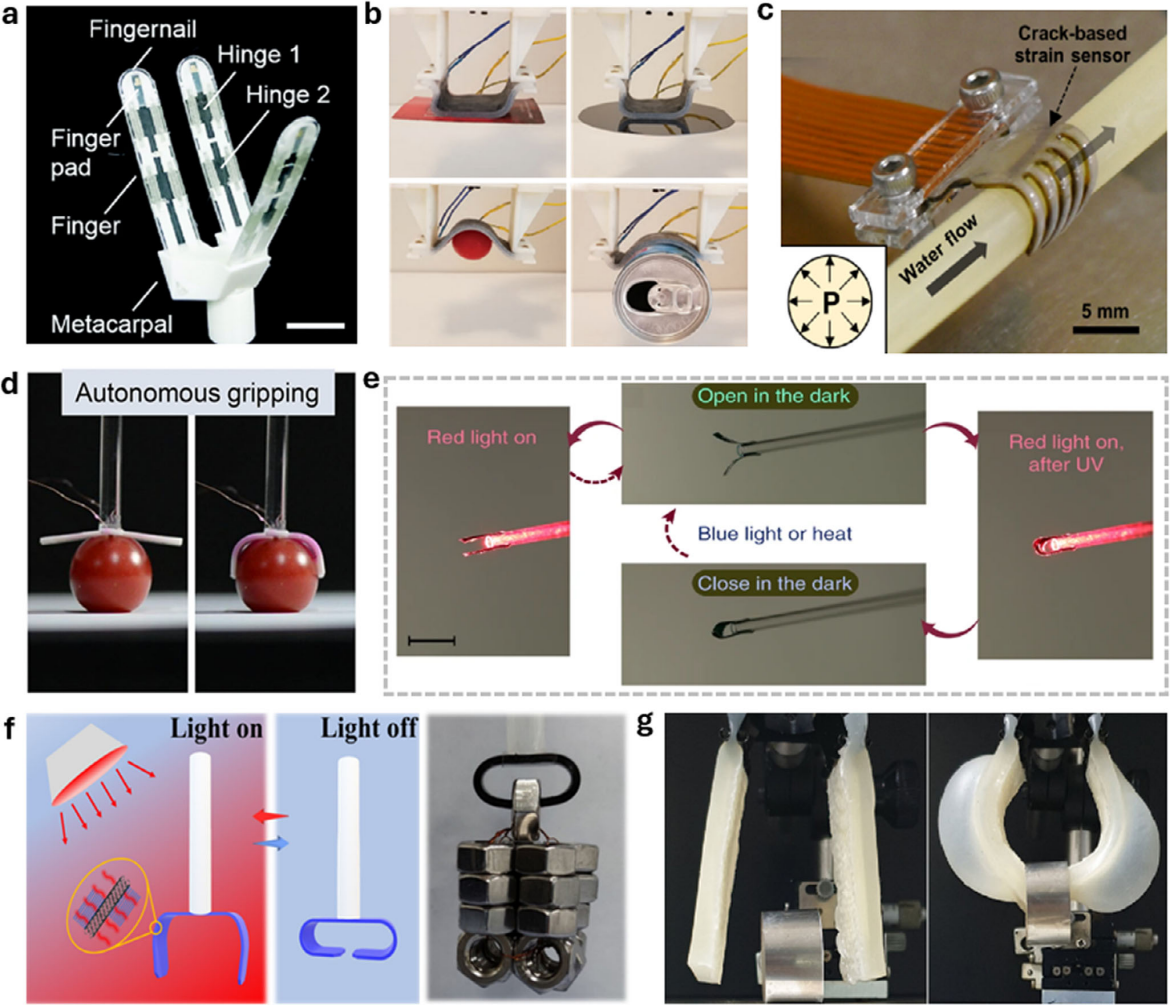
Thermally actuated soft grippers. a) An SMA‐based finger‐like soft robotic gripper with variable stiffness design (Reproduced with permission.^[^ ^202^ ^]^ Copyright 2017, Mary Ann Liebert). Scale bar is 40 mm. b) Printed LM‐based perceptive soft gripper with embodied intelligence (Reproduced with permission.^[^ ^205^ ^]^ Copyright 2022, Mary Ann Liebert). c) A soft robotic gripper with embedded sensors for vital signal sensing (Reproduced with permission.^[^ ^203^ ^]^ Copyright 2021, American Association for the Advancement of Science). d) A LCE gripper with embedded LM as both heaters and strain sensors (Reproduced with permission.^[^ ^206^ ^]^ Copyright 2021, American Chemical Society). e) A reconfigurable micro‐gripper that can be reprogrammed by UV exposure (Reproduced with permission.^[^ ^216^ ^]^ Copyright 2018, Springer Nature). Scale bar is 5 mm. f) An IR light‐triggered soft gripper with capability of lifting 146 times self‐weight (Reproduced with permission.^[^ ^209^ ^]^ Copyright 2022, American Chemical Society). g) A thermal pneumatic soft gripper enabled by liquid‐vapor phase transition (Reproduced with permission.^[^ ^214^ ^]^ Copyright 2021, Mary Ann Liebert).

One of the standout features of soft grippers is their ability to manipulate fragile objects. By conforming to the shape of the object, soft grippers can maintain a secure hold without exerting excessive pressure. To achieve this conformal and delicate manipulation, two main strategies have been adopted — tunable stiffness and embedded sensing (**Table** **2**). For example, a SMA‐based soft gripper composed of three identical fingers has been demonstrated with variable stiffness for adaptive grasping in the low stiffness state and effective holding in the high stiffness state. Each hinge of the robotic finger can achieve a stiffness tunable by a factor of 55 independently (**Figure** **9**a).^[^ ^202^ ^]^ Another gripper showcased not only tunable stiffness but also tunable friction for versatile gripping (Figure 9b).^[^ ^205^ ^]^ The gripper functions by first bringing the pad into contact with an object while it is in a heated and soft state. It then allows the pad to cool and harden, creating a strong adhesive bond before lifting the object. For embedded sensing, Roh et al. reported a soft gripper with embedded crack‐based strain sensors for temperature and pressure measurements on grasped objects (Figure 9c).^[^ ^203^ ^]^ This study illustrated the potential for widespread utility of soft grippers in medical surgeries and healthcare.

**Table 2:** Thermally actuated soft grippers.

	Heating mechanism	Heat source	Thermally responsive materials	Frequency (Hz)	Load	Cooling speed (°C s^−1^)	Gripping features
[202]	Joule heating	Ni‐Cr wires	SMA	n/a	5.5 N	0.109	Tunable stiffness
[17]	Joule heating	AgNWs	PDMS/PI	0.25	n/a	1.21	n/a
[203]	Joule heating	AgNWs	SMA	n/a	0.293N (6400 self‐weight)	n/a	Embedded sensing
[204]	Joule heating	SMA	SMA	0.1	30 N	n/a	n/a
[205]	Joule heating	CB	LMPP	n/a	1.44 N	n/a	Tunable stiffness and adhesion
[206]	Joule heating	LM	LCE	n/a	0.07 N	n/a	Embedded sensing
[183]	Joule heating	Ni‐Cr	LCE	0.05	0.0025 N (210 self‐weight)	3.2	n/a
[162]	Joule heating	Ni‐Cr	Novec7000	0.025	0.265 N (18 self‐weight)	>n/a	n/a
[207]	EMI	Fe_3_O_4_ NPs	water	n/a	31N	0.72	n/a
[208]	EMR	LED light	LCE	n/a	0.000056 N	n/a	n/a
[209]	EMR	IR light	POE/PW	0.0042	0.85 N (146 self‐weight)	n/a	Tunable stiffness and self‐sensing
[210]	EMR	NdFeB	SMP	n/a	0.225 N (64 self‐weight)	n/a	Tunable stiffness
[211]	Fluid Convection	Water	NIPAM	0.125	n/a	n/a	n/a
[212]	Fluid Convection	Air	LCE	n/a	0.02 N	n/a	n/a
[213]	Fluid Convection	Air	SMP	0.025	8.2 N	n/a	n/a
[214]	Fluid Convection	Air	Novec7000	n/a	n/a	n/a	n/a
[55]	Fluid Convection	Air	Ethanol	n/a	0.392 N	n/a	n/a

Moreover, soft grippers have broader industrial applications, such as in automated assembly lines. Figure 9d demonstrates the idea of autonomous gripping. LM circuits printed on the LCE surface were multifunctional, serving both as a resistive heater and as a sensory skin capable of detecting its deformation. These LM circuits enabled biomimetic autonomous actuation in response to mechanical stimuli such as pressure or strain.^[^ ^206^ ^]^

The programmability of soft grippers can be particularly beneficial in various situations. Figure 9e shows a reconfigurable micro‐gripper at different stages. With the combination of UV and temperature stimuli, the gripper can switch between grip‐and‐release mode and grip‐and‐hold mode.^[^ ^216^ ^]^ The programmability can also be achieved using phase transition materials. Taking advantage of tunable reversible deformation and phase transition of a polyolefin elastomer (POE)/paraffin wax actuator, a remotely controlled gripper with high load‐bearing capability (weight‐lifting ratio > 146) has been demonstrated (Figure 9f).^[^ ^209^ ^]^

Other than traditional Joule heating, EMI and EMR heating, fluid convection has also been widely adopted. Figure 9g shows a liquid‐vapor phase change‐enabled thermal‐pneumatic soft gripper.^[^ ^214^ ^]^

#### 5.2.2 Bio‐Inspired Manipulators

Venus flytrap, *Dionaea muscipula*, is a carnivorous plant that gets nutrients from trapping prey between its touch‐sensitive bivalved leaves.^[^ ^217^ ^]^ The mechanism relies on the plant's unique ability to store and release elastic energy that can trigger a snap‐through mechanism. Thermal actuators are known for their slow actuation speed, but by varying the structure as introduced in Section 4.5, Wu et al., created an offset displacement in the traditional bimorph structure (Figure 7a), and adopted the snap‐through instability mechanism to mimic the flytrap's ability to instantly switch between open and closed states.^[^ ^19^ ^]^

Another example is the vine/tendril‐like plant. The climbing vines in particular exhibit helical growth patterns, where they bend, twist, and spiral in search of support and grasp. This motion pattern allows the plant to efficiently explore its surroundings until it encounters a branch or pole. Researchers have developed different methods to mimic this twisting motion, from creating a piecewise control of two different electrothermal actuators to utilizing localized EMR to actuate specific areas of the actuator.^[^ ^165^, ^218^, ^219^ ^]^ A robotic tendril was demonstrated with three different segments with different filament orientations, allowing for sequential deformation of each segment to ensure a symmetric internal coiling, pulling, and firm grasping of the target structure. This biological system can be leveraged for different applications. Hu et al. demonstrated a self‐oscillating tendril fiber actuator and a laser steering system to control the tendril direction. They also developed another design with a high contraction ratio (1750%) and high stress (≈3.4 MPa) LCE tendril fiber actuator.^[^ ^165^, ^219^ ^]^

### 5.3 Biomedical Devices

#### 5.3.1 Assistance, Rehabilitation, and Accessibility

Many researchers, when developing soft actuators for assistive or rehabilitation technologies, have turned to biology for inspiration, in particular, muscles. The natural muscle is a soft biological actuator that demonstrates large stress, high deformability, and high fatigue life, significantly outperforming current robots.^[^ ^224^, ^225^ ^]^ Researchers like Lathers et al., Liu et al., and Tasmin et al. have engineered muscle‐like actuators specifically for use in prosthetics or limb rehabilitation.^[^ ^226^, ^227^, ^228^ ^]^

**Figure 10:**
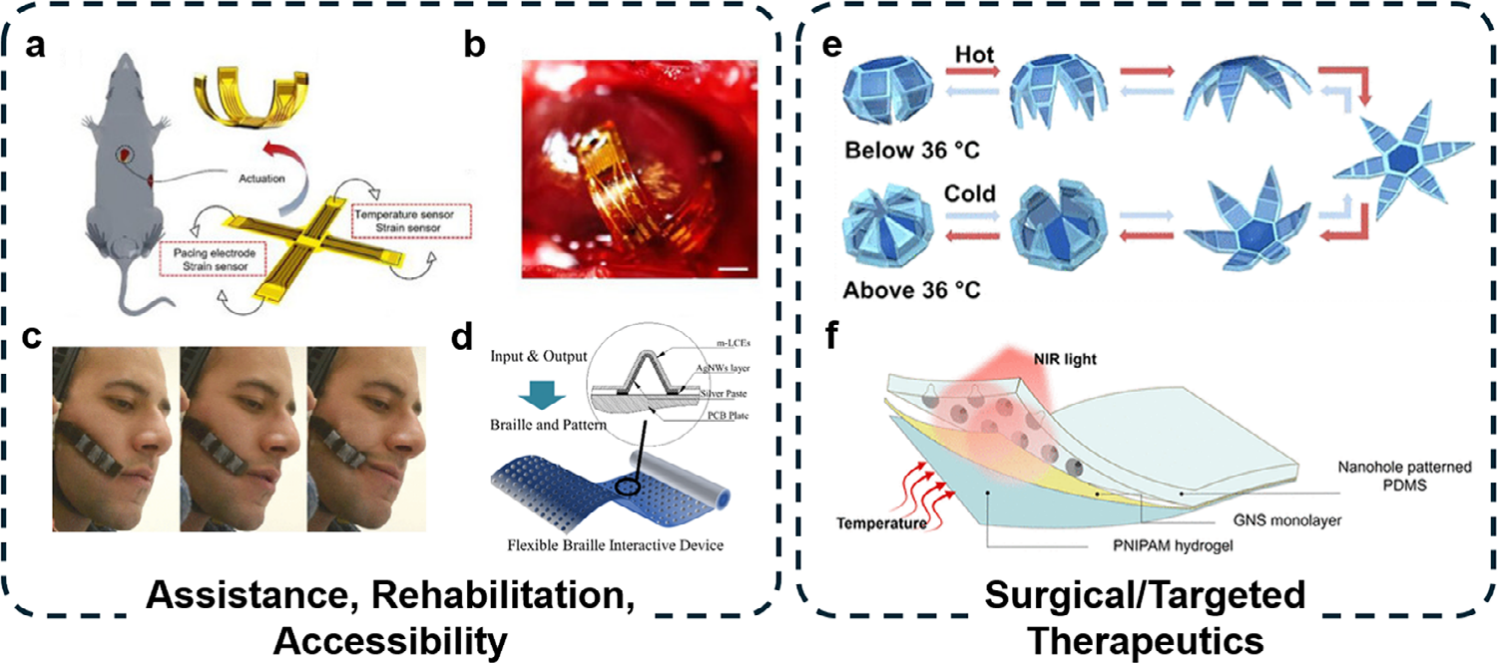
Biomedical Applications of Soft Thermal Actuators. a) Schematic of an unactuated and actuated soft robot with pacing electrode and epicardial sensors inserted into a live mouse. b) Image of an actuated soft robot on a living beating mouse heart.^[^ ^75^ ^]^ Scale bar is 5 mm. (a and b: reproduced with permission.^[^ ^75^ ^]^ Copyright 2024, Springer Nature) c) Image of facial rehabilitation device in action (Reproduced with permission.^[^ ^220^ ^]^ Copyright 2017, IEEE) d) Schematic of flexible braille interactive device (Reproduced with permission.^[^ ^221^ ^]^ Copyright 2022, American Chemical Society. e) Schematic of a thermal gripper in varying temperatures (Reproduced with permission.^[^ ^222^ ^]^ Copyright 2015, American Chemical Society) f) Schematic of layer‐by‐layer Plasmonic AdPatch (Reproduced with permission.^[^ ^223^ ^]^ Copyright 2024, American Chemical Society).

While muscle‐like actuators remain a major topic in thermal actuators, other thermal‐actuator‐based assistive technologies have also been explored for these applications. For example, researchers have developed an untethered, soft robot to enable structural adaptation and dynamic reconfiguration to internal organs to minimize tissue damage.^[^ ^75^ ^]^ **Figure** **10**a shows a robotic patch as an example, composed of a layer of PNIPAAM hydrogel, temperature sensors, a pacing electrode made of Au, and strain sensors made of patterned Au/PI, parylene was used to encapsulate the device. This patch can actively grasp and release a beating heart for both epicardial sensing and pacing. When inserted into the body, the gripper is at the resting state (flat) and upon actuation, wraps onto the surface of a living beating mouse heart (Figure 10b). The gripper can provide electrical stimulation with different voltages, allowing for selective and local pacing of cardiac cells to restore normal heart function.^[^ ^75^ ^]^ Kongahage et al. developed a larger‐scale ventricular assist device, creating a heart sleeve embedded with silver‐coated nylon, coated with a silicon insulation layer. This device uses the contracting behavior of the actuator to mechanically simulate the heart, imitating the motions of a heart to aid patients.

In addition to in‐body assistive devices, a prominent area of soft biomedical devices focuses on external assistive devices, such as prosthetics and assistive grippers. Atalay et al. reported a textile‐based robotic glove to assist users with grasping using enclosed thermally responsive fluids.^[^ ^229^ ^]^ Assistive technologies can also be used for rehabilitation, such as the ASR glove, developed for both hand assistance and rehabilitation using SMAs.^[^ ^230^ ^]^ Figure 10c shows an example that leveraged the key advantage of soft robotics and applied it to a highly sensitive and fragile body part, the face. In this work, embedded SMPs are used as tendons, which are electrothermally controlled with a stretchable heater to replicate and assist with natural motions on the paralyzed side of the face for patients suffering from facial palsy.^[^ ^220^ ^]^

Soft thermal actuators can also be used to promote accessibility by creating interactive braille displays. Figure 10d is an LCE/AgNW‐based flexible interactive display composed of multiple cells.^[^ ^221^ ^]^ Each cell is electrothermally stimulated and allows for the dual perception of the height difference of each cell and the temperature difference for an actuated dot.

#### 5.3.2 Surgery and Targeted Therapeutics

When developing micro‐robots for in‐body applications such as surgery and targeted therapeutics, many uses combine stimuli, the most common being guided by a magnet and triggered thermally. Ji et al. used ferrofluid for targeted tumor therapy, starting with guiding the ferrofluid with a magnet, then using an NIR laser to heat the ferrofluid at a targeted location to destroy the cancer cells.^[^ ^231^ ^]^ Other NIR‐triggered microrobots also allow for shape‐switching for maximum drug delivery^[^ ^232^ ^]^ or real‐time X‐ray imaging during treatment.^[^ ^233^ ^]^ Other actuation techniques include electromagnetically induced drug delivery.^[^ ^234^ ^]^ Micro‐grippers are also developed for in‐body assembly or surgeries.^[^ ^222^, ^235^ ^]^ Figure 10e shows a schematic of a self‐folding thermally actuated soft microgripper made of a poly(N‐isopropylacrylamide‐co‐acrylic acid) hydrogel.^[^ ^222^ ^]^ Iron(III) oxide nanoparticles are embedded into the hydrogel, allowing the gripper to be guided by magnetic fields. This device can be used for surgical excision or pick‐and‐place tasks. The work demonstrated the capture and excision of cells from a live fibroblast cell clump.

Targeted therapeutics include drug‐delivery patches. The most common thermal‐actuated drug delivery patches use thermally responsive hydrogel drug carriers to release the desired treatment for wound dressing or targeted treatment.^[^ ^236^, ^237^ ^]^ Kim et al. designed an octopus‐inspired smart adhesive patch, drawing inspiration from the suction cups that enable octopuses to not only taste, feel, and touch, but also provide them with advanced manipulation and environmental interaction capabilities. The Plasmonic AdPatch incorporated a series of nanocavities analogous to those of the octopus's suction cups, successfully creating a strong adhesion between the smart patch and the wearer's skin.^[^ ^223^ ^]^ Figure 10f shows the layer‐by‐layer design of the patch. Once the temperature increases to 45 °C, the walls of each nano‐hole shrink, causing the internal pressure to increase to achieve a final strong adhesive.^[^ ^223^ ^]^

### 5.4 Interaction and Immersion

Thermal actuators enable soft robots to simulate life‐like behaviors and enhance user experiences. In companion and therapy robots, thermal actuators not only create natural movements like other actuators, but also produce realistic warmth, providing comfort to patients. In virtual reality (VR)/augmented reality (AR) applications, these actuators add a tactile dimension to virtual environments, delivering sensations like warmth or coolness for better immersion. Additionally, in aesthetic applications, thermal actuators enable dynamic changes in form, texture, and color, bringing art installations, sculptures, and even adaptive camouflage to life.

#### 5.4.1 Companion and Therapy

**Figure 11:**
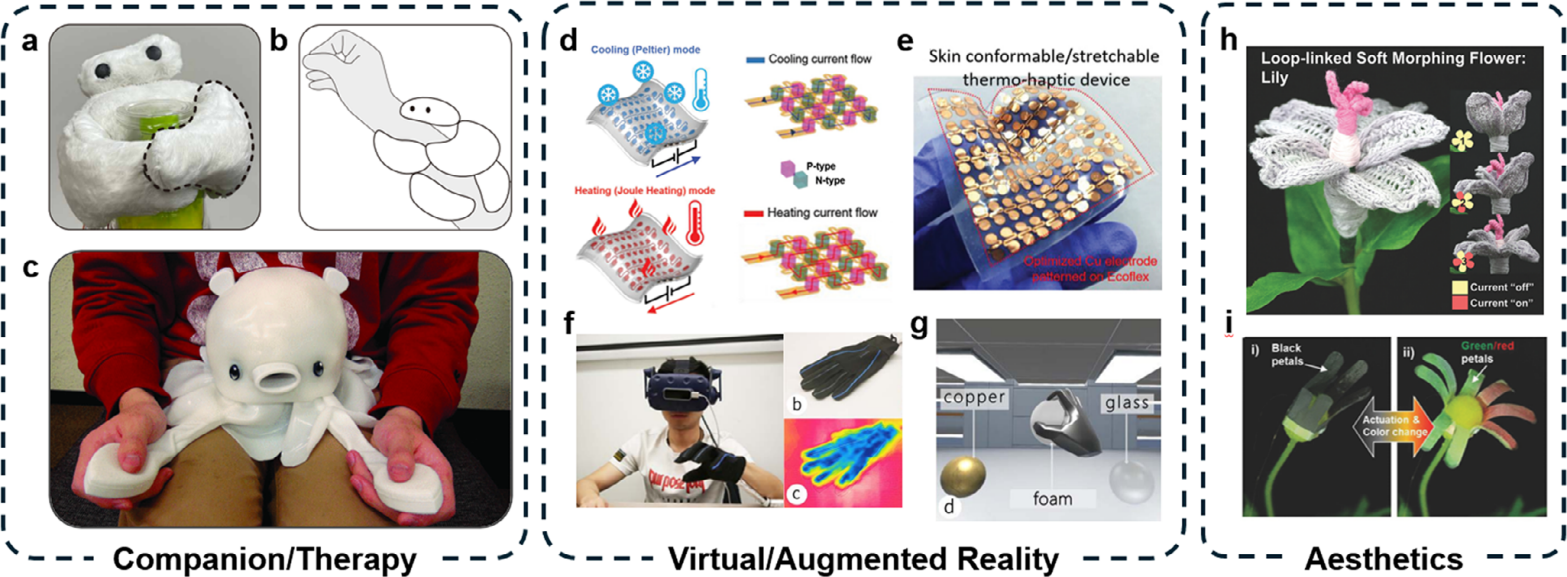
Interaction and Immersion Applications of Soft Thermal Actuators. a) Photograph of Reliebo with an outline where the SMA–soft pneumatic actuator is located.^[^ ^238^ ^]^ b) Schematic of how Reliebo interacts with people. (a and b: Reproduced with permission.^[^ ^238^ ^]^ Copyright 2024, Association for Computing Machinery) c) Photograph of a thermally responsive variable stiffness social robot in use. (Reproduced with permission.^[^ ^239^ ^]^ Copyright 2021, IEEE) d) Schematics of an on‐skin thermo‐haptic device in cooling and heating mode with locations of P‐type and N‐type thermoelectric pellets.^[^ ^240^ ^]^ e) Photograph of the on‐skin thermo‐haptic device highlighting the patterned Cu electrode embedded into the Ecoflex elastomer (d and e: Reproduced with permission.^[^ ^240^ ^]^ Copyright 2020, WILEY‐VCH). f) Images of ThermAirGlove with thermo‐haptic actuators in use, resting, and the corresponding IR image when glove is filled with cold air.^[^ ^241^ ^]^ g) TAGlove application in virtual scene where user is grasping a foam ball (f and g: Reproduced with permission.^[^ ^241^ ^]^ Copyright 2020, WILEY‐VCH) h) An artificial flower enabled by knitted SMA patterns showing movements blooming (Reproduced with permission.^[^ ^242^ ^]^ Copyright 2017, WILEY‐VCH) i) Blooming motion of a color shifting flower composed of anisotropic soft actuators (Reproduced with permission.^[^ ^243^ ^]^ Copyright 2018, WILEY‐VCH).

Companion and therapy robots are playing an increasingly important role in modern society, being used in various fields such as education, healthcare, entertainment, and daily life. They interact with people through different actuation methods, including auditory, optical, and haptic. Thermal actuators improve movement and responsiveness in these robots, enabling them to mimic breathing or body warmth. Yim et al. integrated SMA with a soft pneumatic actuator to improve Reliebo, a hand‐held wearable inflatable robot to alleviate pain and fear,^[^ ^244^, ^245^ ^]^ by enhancing emotional and affective touch^[^ ^238^ ^]^ (**Figure** **11**a–b). The thermo‐pneumatic hybrid actuator offers a wide range of movements from a gentle hold to a squeeze sensation. Figure 11c demonstrates a social robot that offers variable softness by using a thermally responsive hydrogel that changes stiffness depending on the temperature, controlled by both convective fluid flow and electrothermal heaters.^[^ ^239^ ^]^

#### 5.4.2 Virtual and Augmented Reality

In part due to the emergence and popularity of head‐mounted displays (HoloLens, Vive, Oculus, etc), the field of VR/AR is experiencing rapid growth.^[^ ^246^ ^]^ While current VR/AR technologies rely mainly on visual and auditory stimulation to immerse the player, tactile senses play a significant role in daily life. Soft thermal actuators allow the player to receive accurate and realistic sensations as thermal actuators can not only generate stretching, bending, or twisting motions but also simulate temperature changes, making virtual interactions more lifelike.^[^ ^247^ ^]^ Some thermal actuators have demonstrated the use of SMA for skin deformation to improve haptic actuation in VR/AR applications, moving away from the more conventional vibrational motors.^[^ ^248^ ^]^ Researchers are also developing thermo‐haptic devices,^[^ ^240^, ^241^, ^247^, ^249^ ^]^ for example, as shown in Figure 11d–g. Figure 11d is a thermo‐haptic device that can simulate both cooling and heating with a focus on replicating thermal sensations in VR using an Ag‐Ecoflex composite, Cu electrodes, thermoelectric pellets, and PI.^[^ ^240^ ^]^ Figure 11f shows ThermAirGlove, a glove combining both pneumatic and thermal actuation to improve the haptic sensation of object grabbing by providing different temperatures and materials in VR.^[^ ^241^ ^]^ This sensation improved users’ material identification in VR.

#### 5.4.3 Aesthetics

Outside of functionality and safety, soft robotics is aesthetically more favorable for humans because they look and feel more “natural” or move more “naturally”, allowing for more pleasant interactions.^[^ ^250^, ^251^ ^]^ Aesthetic aspects are of critical relevance to developing soft robots that directly interact with humans, impacting how people perceive and understand the robot's behavior.^[^ ^252^ ^]^ Aesthetics also allows for better visual expression, creativity, and interactions in artistic or entertainment settings. In addition to applications in natural movement, soft thermal actuators are found in color‐changing devices and technologies.^[^ ^253^ ^]^ The use of adaptive coloration and shape‐shifting abilities not only promotes the aesthetics of a device but also provides better camouflage, allowing the device to blend seamlessly into its surroundings.^[^ ^254^ ^]^

Flowers represent a unique example in aesthetics, particularly in their ability to simultaneously or sequentially bloom and fold.^[^ ^243^, ^255^, ^256^ ^]^ The flower‐like movement allows for the creation of deployable systems that can unfold/expand in confined spaces. Figure 11h integrates SMA into knit patterns to mimic the natural movements of a flower blooming.^[^ ^242^ ^]^ Figure 11i highlights the color‐changing of a multi‐functional thermal actuator.^[^ ^243^ ^]^ Eight color‐shifting anisotropic actuators are initially flat and black, and upon electrothermal actuation, each petal heats up, causing the petal to color bright green/red and bend outward.

## 6 Conclusion and Outlook

With the advent of novel functional materials and innovative structural designs, soft thermal actuators and robotics have garnered unprecedented attention from both academic and industrial sectors. ​In this paper, we categorize four fundamental heating mechanisms, including heat generation mechanisms–Joule heating, EMI, EMR; and heat transfer mechanisms–fluid convection. Within each category, we conduct a thorough examination of material selection, analyzing the advantages and disadvantages of various heating materials and thermally responsive materials.

We also discuss structural designs that are aimed at enhancing actuation performance. The incorporation of structural designs such as bending structures, twisting or spiral geometries, and other 3D deformable designs has facilitated a wide range of deformation modes tailored for specific functions. In addition, the implementation of instability designs significantly improves the actuation speed and output force during thermal actuation. Effective thermal management—self‐sensing capabilities and temperature regulation—are discussed to enhance both precision and efficiency in thermal actuation.

For the application sections, we investigated four primary categories: locomotion robots, soft grippers, biomedical devices, and interaction and immersion devices. We meticulously compare representative examples from each category, providing insights into the respective advantages and limitations of various actuators and robotic systems.

Bioinspiration, as discussed throughout the review, plays a critical role in materials engineering, structural design, and applications. By drawing insights from biological materials, researchers can develop innovative materials with enhanced properties, such as improved flexibility, stretchability, and strength. In structural design, bioinspiration enables the creation of novel structures that mimic the elegant, yet efficient and powerful ones found in nature. These biologically inspired approaches have far‐reaching applications across various fields, including robotics, medical devices, rehabilitation, and VR/AR.

The future of soft thermal actuators and robotics continues to face a range of significant challenges to their effectiveness and broader application. Major challenges such as inherently low actuation speed, unsatisfactory energy efficiency, and a lack of intelligent feedback control must be addressed to realize the full potential of these innovative technologies.

One of the foremost challenges is the relatively low actuation speed of soft thermal actuators. The advancement in actuation speed relies heavily on the synergistic development of materials science and structural designs. For instance, recent endeavors in utilizing advanced materials such as liquid crystal elastomers (LCEs) have shown promising results in achieving higher actuation speeds due to their unique thermally responsive molecular alignment and phase transition properties.^[^ ^52^, ^257^ ^]^ Additionally, structural designs involving snap‐through instability and bistable mechanisms have demonstrated great potential in increasing speed by storing and rapidly releasing elastic energy.^[^ ^19^, ^182^ ^]^ Such mechanisms can overcome the inherent slowness of thermal diffusion. Collaborative efforts that integrate innovative material with mechanics‐based design principles could yield significant breakthroughs in the speed and responsiveness of soft robots, especially thermally actuated ones operating in time‐sensitive or high‐frequency scenarios.

Energy efficiency remains a critical challenge for thermally actuated soft robotics. Current designs often struggle with energy losses attributable to heat dissipation into the environment, which not only reduces performance but also limits portability and operational duration. One promising approach to improving energy efficiency is the integration of temperature sensors and control circuits that provide real‐time thermal feedback, allowing for smart ON/OFF control of heating elements and minimizing unnecessary energy expenditure.^[^ ^258^, ^259^ ^]^ Bioinspired strategies offer another avenue; for instance, reptiles can dynamically modulate blood flow to the skin to retain or dissipate heat, an elegant and efficient thermal regulation strategy. By mimicking these biological processes, researchers can design systems that manage thermal energy more effectively. Additionally, the integration of thermoelectric materials or heat recovery units to recapture and reuse waste heat could significantly enhance overall efficiency, making thermally actuated soft robots more viable for long‐term or autonomous use.

The systematic integration of sensing technologies into thermally actuated soft robots also presents a significant challenge.^[^ ^260^, ^261^ ^]^ At present, these robots predominantly rely on preprogrammed control or basic environmental stimuli, limiting their adaptability in complex or dynamic settings. To unlock the full potential of these systems, it is crucial to achieve high‐level integration with advanced sensing technologies, such as flexible temperature sensors, strain gauges, or optical feedback systems, alongside onboard data processing units.^[^ ^262^ ^]^ Real‐time feedback control, enabled by such sensors, could allow thermal actuators to modulate their response based on precise environmental or internal conditions, enabling more adaptive, stable, and efficient operation. This kind of sensing‐actuation feedback loop, inspired by biological proprioception and reflex pathways, could substantially improve the autonomy and robustness of soft thermal robots.

The integration with artificial intelligence (AI) could further revolutionize the field, allowing thermally actuated soft robots not only to function autonomously but also to learn from and adapt to their environment.^[^ ^263^ ^]^ AI algorithms, particularly those based on reinforcement learning and neural networks, could process multisensory data streams to make decisions, optimize actuation timing, and adapt motion patterns in real time.^[^ ^264^ ^]^ For example, in complex environments such as within the human body or disaster zones, AI‐equipped robots could autonomously navigate and adapt based on changes in temperature, pressure, or obstacles, much like how animals adjust to dynamic conditions. This level of intelligence could greatly expand the applicability of soft thermal robots to tasks requiring high precision and adaptability, such as minimally invasive surgery, targeted drug delivery, search and rescue missions, or even interactive assistive technologies for individuals with disabilities.

Beyond core engineering challenges, application‐driven contexts such as therapy, virtual reality, and aesthetic interaction introduce unique hurdles. In therapeutic and wearable systems, thermal actuators must prioritize user safety, skin compatibility, and thermal comfort over extended use. Immersive interfaces require compact, fast‐responding actuators capable of delivering finely controlled, local heating and cooling without perceptible lag. Meanwhile, aesthetic and expressive applications require actuators that are lightweight, visually adaptable, and capable of repeated complex deformation without fatigue or degradation. Meeting these needs will involve advances in user‐centered design, material durability, and novel fabrication strategies that balance performance, usability, and long‐term human interaction.

## Conflict of Interest

The authors declare no conflict of interest.
